# Exosomal miR-663b from “M1” macrophages promotes pulmonary artery vascular smooth muscle cell dysfunction through inhibiting the AMPK/Sirt1 axis

**DOI:** 10.18632/aging.204690

**Published:** 2023-05-04

**Authors:** Honghong Ma, Yang Yu, Lirong Mo, Qian Chen, Hui Dong, Yan Xu, Bing Zhuan

**Affiliations:** 1 Department of Respiratory Medicine, People’s Hospital of Ningxia Hui Autonomous Region, Yinchuan 750000, Ningxia, China; 2 Department of Respiratory Medicine, Third Clinical Medical College, Ningxia Medical University, Yinchuan 750000, Ningxia, China; 3 College of Traditional Chinese Medicine, Ningxia Medical University, Yinchuan 750000, Ningxia, China; 4 General Hospital of Ningxia Medical University, Yinchuan 750000, Ningxia, China

**Keywords:** miR-663b, exosomes, macrophages, AMPK pathway, pulmonary artery vascular smooth muscle cells

## Abstract

Background: Inflammatory mediators from macrophages are proven to be involved in pulmonary vascular remodeling in pulmonary hypertension (PH). Here, this study intends to explore the mechanism of “M1” macrophage-derived exosomal miR-663b in pulmonary artery smooth muscle cells (PASMCs) dysfunctions and pulmonary hypertension.

Methods: Hypoxia-treated PASMCs were utilized for constructing an *in-vitro* pulmonary hypertension model. THP-1 cells were treated with PMA (320 nM) and LPS (10 μg/mL) + IFN-γ (20 ng/ml) for eliciting macrophage “M1” polarization. Exosomes derived from “M1” macrophages were isolated and added into PASMCs. The proliferation, inflammation, oxidative stress, and migration of PASMCs were evaluated. RT-PCR or Western blot examined the levels of miR-663b and the AMPK/Sirt1 pathway. Dual luciferase activity assay and RNA pull-down assay were carried out for confirming the targeted association between miR-663b and AMPK. An *in-vivo* PH model was built. Macrophage-derived exosomes with miR-663b inhibition were used for treating the rats, and alterations of pulmonary histopathology were monitored.

Results: miR-663b was obviously up-regulated in hypoxia-elicited PASMCs and M1 macrophages. miR-663b overexpression boosted hypoxia-induced proliferation, inflammation, oxidative stress, and migration in PASMCs, whereas miR-663b low expression resulted in the opposite situation. AMPK was identified as a target of miR-663b, and miR-663b overexpression curbed the AMPK/Sirt1 pathway. AMPK activation ameliorated the damaging impact of miR-663b overexpression and “M1” macrophage exosomes on PASMCs. *In vivo*, “M1” macrophage exosomes with miR-663b low expression alleviated pulmonary vascular remodeling in pulmonary hypertension rats.

Conclusion: Exosomal miR-663b from “M1” macrophage facilitates PASMC dysfunctions and PH development by dampening the AMPK/Sirt1 axis.

## INTRODUCTION

Pulmonary hypertension, a pathophysiological condition of aberrantly heightened pulmonary artery pressure triggered by several known or unknown factors, is characterized by shortness of breath, fatigue, angina, and other symptoms [1]. The pathogenesis of pulmonary arterial hypertension (PAH) may encompass various factors like nitric oxide signaling, vascular endothelial dysfunction, oxidative stress, and inflammation [2]. Pulmonary vascular smooth muscle cells are the primary ingredients of pulmonary vessels, and their proliferation and apoptosis resistance would contribute to lumen stenosis and wall stiffness in pulmonary vessels, thus boosting pulmonary hypertension occurrence and development [3]. Current treatment strategies cannot effectively deter or invert pulmonary hypertension development [4]. Therefore, a probe into new strategies for preventing and inverting PAH is desperately needed.

Macrophages have been found with dual roles in pulmonary diseases, whether or not maintaining lung homeostasis and protecting against inflammation [5]. Alveolar macrophages are approximately 95% of airspace leukocytes and affect PAH through the synthesis and release of various inflammatory mediators [6]. A recent study shows that during PAH development, macrophages interact with PASMCs through CCR2/CCR5 to initiate and amplify PASMC migration and proliferation [7]. Notably, macrophages have different polarization states, which range from a proinflammatory state (also known as classically activated or M1 macrophages) to a protective state (also known as alternatively activated or M2 macrophages) [8]. Alternatively activated (M2+ or M1+) macrophages have been associated with the development of PAH [9]. Exosomes, characterized by a cup-shaped morphology and diameters ranging from 50 nm to 150 nm, have been identified as essential mediators of macrophages in different diseases [10–11]. Exosomes derived from macrophages contain rich kinds of proteins, lipids, and nucleic acids (DNA, mRNA, and miRNA) [12]. Exosomal-enclosed microRNAs display a regulatory function in pulmonary hypertension development [13–14]. Recently, our study group has found that miR-4640-5p serves as a potential diagnostic biomarker and therapeutic target for COPD-PH. miR-4640-5p inhibition improves proliferation and migration disorders of PASMC via regulating mTOR/S6 signaling [15]. MicroRNA-663b (miR-663b), a member of the microRNA family, has been demonstrated to be substantially up-regulated in the plasma of patients suffering from acute myocardial infarction (AMI) and works as an underlying predictor of AMI [16]. miR-663b had enhanced expression in cervical cancer tissue and exosomes. Cervical cancer cells exosomal miR-663b aggravated angiogenesis of vascular endothelial cells and tumor growth [17]. Nevertheless, the functions of miR-663b in exosomes derived from macrophages in PAH remain obscure.

Adenosine monophosphate-activated protein kinase (AMPK), an AMP-dependent protein kinase, is a critical molecule for modulating biological energy metabolism, exerting a crucial function in the pathogenesis of pulmonary vascular remodeling [18]. For example, Κ-Opioid Receptor Stimulation weakens AMPK phosphorylation and initiates mTOR to curb PASMC proliferation and autophagy and augment apoptosis, hence relieving hypoxic pulmonary hypertension [19]. Sirtuin1 (Sirt1), a NAD-dependent class III histone deacetylase (HDAC), deacetylates cellular signaling molecules and chromatin histones to modulate inflammation, apoptosis, anti-stress, and DNA damage repair [20]. Resveratrol activates Sirt1 to suppress hypoxia-induced PASMC proliferation and facilitate PASMC apoptosis [21]. Sirt1 is a downstream target gene of AMPK [22]. Activating the AMPK-Sirt1 pathway shows protective effects in lung injury. For instance, in an acute lung injury (ALI) model induced by lipopolysaccharide (LPS), Irisin initiates the AMPK/Sirt1 pathway to dampen inflammation and apoptosis, thus alleviating alveolar epithelial barrier dysfunction [23].

Our previous research has revealed that hypoxia-induced pulmonary artery smooth muscle dysfunction can be mitigated after improving mitochondrial morphology and ER stress [24]. Presently, we tried to figure out the role of miR-663b and “M1” macrophages in PASMC proliferation, inflammation, oxidative stress, and migration. miR-663b level was substantially heightened in M1-polarized macrophages, exosomes-derived form “M1” macrophages, and the pulmonary hypertension model. Protein kinase AMP-activated catalytic subunit alpha 2 (PRKAA2), a catalytic subunit of AMPK, is identified as a direct target of miR-663b. Thus, we speculated that miR-663b facilitated hypoxia-elicited PASMC damage potentially via targeting the AMPK/Sirt1 signaling pathway.

## MATERIALS AND METHODS

### Clinical samples

Blood samples from 22 patients diagnosed with chronic obstructive pulmonary disease (COPD)-PH and 22 healthy control donors from the People’s Hospital of Ningxia Hui Autonomous Region were collected. The serum was isolated and total RNA was extracted using TaqMan ABC miRNA Purification Kit (Thermo). The clinical parameters, including FEV1% Pred, FEV1/FVC ratio and pulmonary artery systolic pressure (PASP), were collected. The COPD-PH patients included 14 men and 8 women, and their age ranges from 41 to 76 years old (59 ± 10.3 years old). The 22 healthy control donors included 12 men and 10 women, and their age ranges from 38 to 75 years old (56 ± 8.9 years old). The study protocols were approved by the research ethics committee of the People’s Hospital of Ningxia Hui Autonomous Region and were conducted in accordance with the principles expressed in the Declaration of Helsinki. Informed consent was obtained from all participants.

### Culture and treatment of cells

American Type Culture Collection (ATCC, Rockville, MD, USA) was the supplier of PASMCs and THP-1 cells. An RPMI-1640 medium supplemented with 10% fetal bovine serum (FBS; Gibco, Carlsbad, CA, USA), 100 U/mL penicillin, and 0.1 mg/mL streptomycin (Sigma-Aldrich, St Louis, MO, USA) was taken for the culture of the cells, and the procedure was implemented with the use of an incubator (5% CO_2_, 37°C). Then, 0.25% trypsin (Gibco) was used for digesting the cells. PMA (320 nM) and LPS (10 μg/mL) plus IFN-γ (20 ng/ml) treated THP-1 cells for 24 hours for eliciting macrophage M1 polarization, and M1 macrophages were grown in a 10 mL serum-free medium. PASMCs were treated under hypoxic conditions. The hypoxia group was kept in a hypoxic incubator with 5% CO_2_, 5% O_2_, and 90% N_2_ at 37°C for 24 hours, whereas the control group was placed in a normal incubator with 5% CO_2_, 21% O_2_, and 74% N_2_ for 24 hours.

### Cell transfection

PASMCs amid logarithmic growth were seeded onto 6-well plates (density: 5 × 10^5^/well). Upon 50%–60% confluence, Lipofectamine 2000 transfection reagent (Invitrogen, Carlsbad, CA) transfected miR-NC, miR-663b mimics, miR-in, and miR-663b inhibitors into PASMCs as instructed. Forty-eight hours later, we harvested the cells and examined the efficiency of transfection. Genepharma (Shanghai, China) provided us with miR-663b mimics (50 nM) and their negative control as well as miR-663b inhibitors (100 nM) and their negative control.

### Isolation of exosomes

As M0 and M1 macrophages attained 70–80% confluence, a medium supplemented with 5% exosome-depleted fetal bovine serum (System Biosciences, Inc., Palo Alto, CA, USA) was taken to substitute the original one for a 48-hour culture. The exosomes from the culture medium of M0 and M1 macrophages were separated through ultracentrifugation. The isolated exosomes were identified by scanning electron microscope (SEM).

### The co-culture model

Transwell chambers with 6 wells (diameter: 0.4 μm) were adopted to engineer an interplay model. PASMCs were inoculated into the lower compartment (2 × 10^5^ cells), while M1 macrophages, M0, and M1 macrophage exosomes (6 × 10^5^ cells) were seeded onto the upper room. Later, the co-culture model was placed in an incubator at 37°C for 24 h of culture, followed by the replacement of the medium. After 72 hours, we collected the cells in the lower compartment for the following steps.

### CCK-8 assay

PASMCs, inoculated onto 96-well plates (density: 2 × 10^3^/well), were cultured for 24 hours and then cultured in an incubator for another 4 hours with 10 μL Cell Counting Kit-8 (CCK-8) solution (Beyotime Biotechnology, Shanghai, China) given to each well. A microplate reader (Thermo Fisher Scientific, Shanghai, China) gauged the absorbance value at 450 nm.

### Determination of ROS levels

DCFH-DA/H2DCFDA-Cellular ROS Assay Kit (ab113851, Abcam) confirmed ROS levels in PASMCs. DCFH-DA is a fluorogenic dye adopted for measuring the activities of hydroxyl, peroxyl, and other reactive oxygen species (ROS) within cells. DCFH-DA spread into the cells and was hydrolyzed into nonfluorescent 2′, 7′-dichlorofluorescein (DCFH). Non-fluorescent DCFH in the cells was oxidized to highly fluorescent DCF by the generated ROS. Following 12 hours culture, the cells were harvested and exposed to a serum-free medium incorporating 10 μM DCFH-DA. They were incubated for 20 minutes in darkness at 37°C, and then a fluorescence microscope examined and photographed DCF fluorescence. The relative level of fluorescence was quantified through the fluorescence spectrophotometer (F4000; Hitachi Software Engineering, Yokohama, Japan).

### MDA and 4-HNE level determination

PASMCs were seeded onto 6-well plates. We harvested the cell supernatant and examined MDA and 4-HNE contents in the cells with the use of commercial kits (Beyotime Institute of Biotechnology Shanghai, China) as per the supplier’s recommendations. A spectrophotometer was taken for analysis.

### Transwell assay

A medium (cell density: 2 × 10^4^/mL) without any serum was utilized to prepare single-cell suspension with PASMCs in the logarithmic growth stage, and 100 μL of the suspension was applied to Transwell chambers (Millipore, Billerica, MA, USA). The culture plates were filled with 600 μL medium and 10% FBS. Later, the Transwell chambers and culture plates were kept in an incubator for 12 hours. PBS was utilized to flush the chambers 3 times, and cotton swabs were adopted to remove the cells on the matrigel and in the upper compartment. Cells that migrated were immobilized with 4% paraformaldehyde for 20 minutes and dyed with crystal violet for 15 minutes. A microscope was taken to monitor the migrated cells.

### Dual luciferase reporter assay

Dual-Luciferase Reporter Assay System (Promega, Fitchburg, WI, USA) substantiated the targeted association between miR-663b and AMPK 3′UTR. Put simply, the wild-type or mutant luciferase reporter plasmid of AMPK 3′UTR was transfected into 293T cells and was transfected together with miR-663b mimics or miR-NC. Subsequent to a 48-hour culture, we discarded the medium. PBS flushed the cells. Cell lysates were given for lysing the cells. At last, the cells were oscillated on a shaker at room temperature (RT) for 10 minutes and centrifuged at 3000 g for 5 minutes. With the supernatant harvested, we gauged the luciferase activity.

### RNA pull-down assay

RNA pull-down assay was conducted for verifying the interaction between miR-663b and AMPK. PASMCs were transfected with the biotin-labeled miR-663b mimic, biotin-labeled mutated miR-663b and negative control. 48 hours after the transfection, the cells were harvested and added with lysis buffer. The lysate was harvested and added to the Dynabeads™ M-280 streptavidin magnetic beads (Invitrogen, Carlsbad, CA, USA). Following incubation for 30 min at room temperature, the retrieved supernatant was detected by qRT-PCR for detecting AMPK mRNA enrichment.

### The construction of the pulmonary hypertension rat model

Twenty-eight male Sprague-Dawley rats (aged 6~8 weeks, 200~250 g in weight) were supplied by the Animal Center of the Experimental Animal Center of Ningxia Medical University (Approval number: SYXK(Ning) 2020-0001). The Institutional Animal Care and Use Committee of the People’s Hospital of Ningxia Hui Autonomous Region authorized all experiments. The rats were randomly distributed to four groups (10 for each): Sham, PH, PH+M1^miR-in^-Exo, and PH+M1^miR-663b-in^-Exo. The rats in the Sham group were reared under atmospheric pressure for 28 days (approximately 718 mmHg, PaO_2_ about 150.6 mmHg), and those in the PH group were kept in a chamber with low pressure and hypoxia (pressure reduced to 380 mmHg, and PaO_2_ relatively lowered to 79.6 mmHg) for 28 days. All animals were reared under a 12-hour light/dark cycle and allowed to access food and water at will. The room temperature was kept at 25°C. From the 30th day, M1 macrophage exosomes (20 μg) began to be used to treat the rats in the PH+M1^miR-in^-Exo group and the PH+M1^miR-663b-in^-Exo group, which lasted for 7 days. At last, the rats were euthanized with gradually increasing CO_2_ and then executed.

### Western blot

The RIPA lysates (Sigma-Aldrich, Shanghai, China) were adopted to extract the total protein out of PASMCs and the rat lung tissues. Following protein denaturation, 30 μg of the protein samples were isolated through 10% SDS-PAGE gel electrophoresis and loaded onto polyvinylidene fluoride membranes (Millipore, Bedford, MA, USA). 5% skimmed milk sealed the non-specific antigen at RT for one hour. The primary antibodies (diluted with a blocking solution) utilized for the overnight incubation (4°C) of the membranes encompassed Anti-iNOS (Abcam, 1:1000, ab178945, Shanghai, China), Anti-COX2 (Abcam, 1:1000, ab62331), Anti-Nrf2 (Abcam, ab62352, 1:1000), Anti-Trx-1 (Abcam, ab26320, 1:1000), Anti-HO-1 (Abcam, ab68477, 1:2000), Anti-AMPK (Abcam, ab32112, 1:1000), Anti-Sirt1 (Abcam, 1:1000, ab32441), and Anti-GAPDH (Abcam, 1:1000, ab9485). Later, Tris-buffered saline and Tween 20 (TBST) flushed the membranes 4 times (8 minutes each). The secondary antibody goat anti-rabbit IgG (Abcam, 1:2000, ab205718) was given for 90-minute incubation (RT). ECL was harnessed to observe the protein bands.

### RT-PCR

TRIzol reagent (Invitrogen) extracted the total RNA from macrophages and their exosomes, PASMCs, and the rat serum and lung tissues, and complementary DNA (cDNA) synthesis was implemented. The MicroRNA reverse transcription kit (Roche, Shanghai, China) was adopted for cDNA synthesis as instructed. In real-time polymerase chain reaction (RT-PCR), GAPDH was taken as the internal parameter of TNF-α, IL-1β, and IL-6, while U6 was regarded as that of miR-663b. RT-PCR was carried out on the ABI7500 real-time PCR system. The conditions for reaction included: 30 seconds of pre-denaturation at 94°C, 25 seconds of denaturation at 94°C, 30 seconds of annealing at 56°C, and 20 seconds of extension at 72°C. The primer sequences are detailed below: miR-663b: forward 5′-CGCTAACAGTCTCCAGTC-3′, reverse 5′-GTGCAGGGTCCGAGGT-3′; TNF-α: forward 5′-CCTCTCTCTAATCAGCCCTCTG-3′, reverse 5′-GAGGACCTGGGAGTAGATGAG-3′; IL-1β: forward 5′-TCTCGCAGACAGCACATCA-3′, reverse 5′-CACACACCAGCAGGTTAT-3′; IL-6: forward 5′-TGGGAAATCGTGGAAATGAG-3′, reverse 5′-CTCTGAAGGACTCTGGCTTTG-3′; U6: forward 5′-CTCGCTTCGGCAGCACA-3′, reverse 5′-AACGCTTCACGAATTTGCGT-3′; GAPDH; forward 5′-GTGGACATCCGCAAACAC-3′, reverse 5′-AAAGGGTGTAACGCAACTA-3′.

### Hemodynamics

Following 4 weeks’ exposure to hypoxia, 4.8% tribromoethanol (7.5 ml/kg) was intraperitoneally transfused into the rates for anesthetization. A polyethylene catheter connected to the transducer was inserted into the right ventricle (RV) via the right jugular vein. Then, the Power Lab software (ADI Instruments) was introduced to record the right ventricular systolic pressure (RVSP). Later, the blood samples, lungs, and hearts of the rats were harvested. With the right ventricle and the left ventricle plus septum (LV+S) collected, the ratio of RV/(LV+S) was worked out and employed as the indicator of right ventricular hypertrophy. The lung tissues were severed into slices (4 mm in thickness) and kept in 4% paraformaldehyde solution for 72 hours. The rat blood samples were subjected to centrifugation, and the serum was isolated. The remaining lung tissues and serum were maintained at −80°C in preparation for the following tests.

### HE staining and Masson staining

At 25°C, 4% paraformaldehyde immobilized the lung tissues for 24 hours. Gradient ethanol was taken for dehydration. Next, the tissues were embedded in paraffin and severed into slices (4 μm thick). After being dewaxed with xylene, the slices were subjected to gradient hydration and hematoxylin-eosin (HE) staining: five minutes of staining with 0.5% hematoxylin at 25°C and 1 minute with 0.5% eosin. Alcohol of gradient concentrations was utilized for hydration, and xylene was taken for dehydration. Masson staining was conducted using Masson's Trichrome Stain Kit (Cat.No. G1340, Solarbio, Beijing, China). All steps were referred to the instructions of the producer. The photos were taken employing an inverted optical microscope (magnification, ×400; Olympus, BX51) under white light.

### Immunohistochemistry

At 25°C, 4% paraformaldehyde immobilized the lung tissues for 24 hours. Gradient ethanol was taken for dehydration. Next, the tissues were embedded in paraffin and severed into slices (4 μm thick). After that, the tissues received deparaffinization by immersing them in xylene or other clearing solutions to remove the paraffin. The tissue sections were hydrated by soaking them in a series of graded alcohols and received antigen retrieval by heat. 5% BSA was used for blocking the sections. The primary antibody of anti-α SMA (1:200, ab247668, Abcam) is incubated with the tissue sections. After tissue washing, Goat Anti-Rabbit IgG H&L (HRP) preadsorbed (ab7090, 1:200, Abcam) secondary antibody is incubated with the tissue sections. The photos were taken employing an inverted optical microscope (magnification, ×400; Olympus, BX51) under white light.

### Analysis of statistics

SPSS 24.0, a statistical software (SPSS Inc, Chicago, IL, USA), was involved in the analysis of data. The outcomes were represented as mean ± SD. An independent student’s *t*-test contrasted two groups, while One-way ANOVA contrasted multiple groups. If *P* < 0.05, statistical significance could be recognized.

### Data availability statement

The data sets used and analyzed during the current study are available from the corresponding author on reasonable request.

## RESULTS

### miR-663b presented a high expression in M1 macrophages and the *in-vivo* and *in-vitro* pulmonary hypertension models

**Figure 1:**
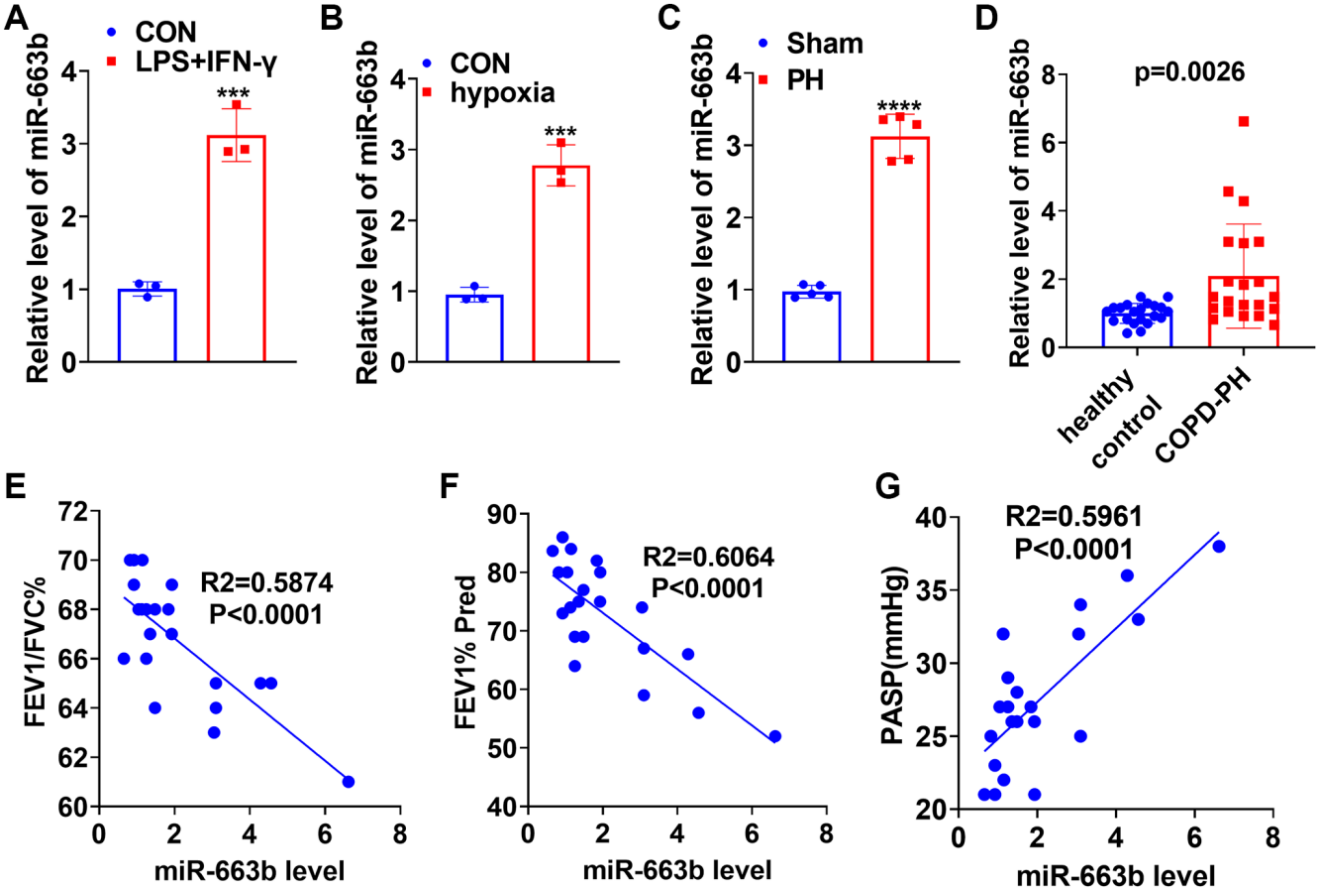
**miR-663b presented a high expression in M1 macrophages as well as in the *in-vivo* and *in-vitro* pulmonary hypertension models.** (**A**) LPS (10 μg/mL) plus IFN-γ (20 ng/ml) treated THP-1 cells for 24 hours to elicit macrophage M1 polarization. RT-PCR determined miR-663b expression in M1 macrophages. (**B**) Hypoxia-treated PASMCs in order to build an *ex-vivo* pulmonary hypertension model. RT-PCR confirmed miR-663b expression in PASMCs treated with hypoxia. *N* = 3. ^***^P < 0.001 (vs. CON). (**C**) A pulmonary hypertension rat model induced by hypoxia was constructed, with RT-PCR conducted to check miR-663b expression in the rat serum. (**D**) RT-PCR was conducted to detect miR-663b expression in the serum of COPD-PH patients (*n* = 22) and health controls (*n* = 22). (**E**–**G**). Pearson linear regression analysis was used for analyzing the associations of serum miR-663b level with FEFV1/FVC% (**E**), FEV1% Pred (**F**) and PASP (mmHg) (**G**). *N* = 5. ^***^P < 0.001, ^****^P < 0.0001 (vs. CON or Sham).

To confirm the expression features of miR-663b in M1 macrophages, we treated THP-1 cells with LPS (10 μg/mL) plus IFN-γ (20 ng/ml) for 24 hours to elicit macrophage M1 polarization. RT-PCR data displayed that in contrast with the CON group, the profile of miR-663b was elevated in macrophages (Figure 1A). For the purpose of a probe into the expression features of miR-663b in the context of pulmonary hypertension, we treated PASMCs with hypoxia to build an *in-vitro* pulmonary hypertension model. The RT-PCR result exhibited that by contrast to the CON group, there was an increase in miR-663b expression in hypoxia-treated PASMCs (Figure 1B). Moreover, a hypoxia-elicited pulmonary hypertension rat model was set up, with RT-PCR implemented to measure miR-663b expression in the rat serum. It turned out that vis-à-vis Sham, the miR-663b level in the serum of the pulmonary hypertension rats was heightened (Figure 1C). We collected serum samples from 22 patients with COPD pulmonary arterial hypertension (IPAH) and 22 healthy controls (HC). miR-663b level was detected and we found that serum miR-663b level was significantly elevated in patients with COPD-PH compared with that of healthy controls (Figure 1D), and higher miR-663b level was significantly associated with the severity of COPD-PH patients (Figure 1E–1G). All these findings revealed that miR-663b presented a high expression in M1 macrophages and in the *in-vivo* and *ex-vivo* models of pulmonary hypertension.

### miR-663b overexpression contributed to PASMC dysfunction

**Figure 2:**
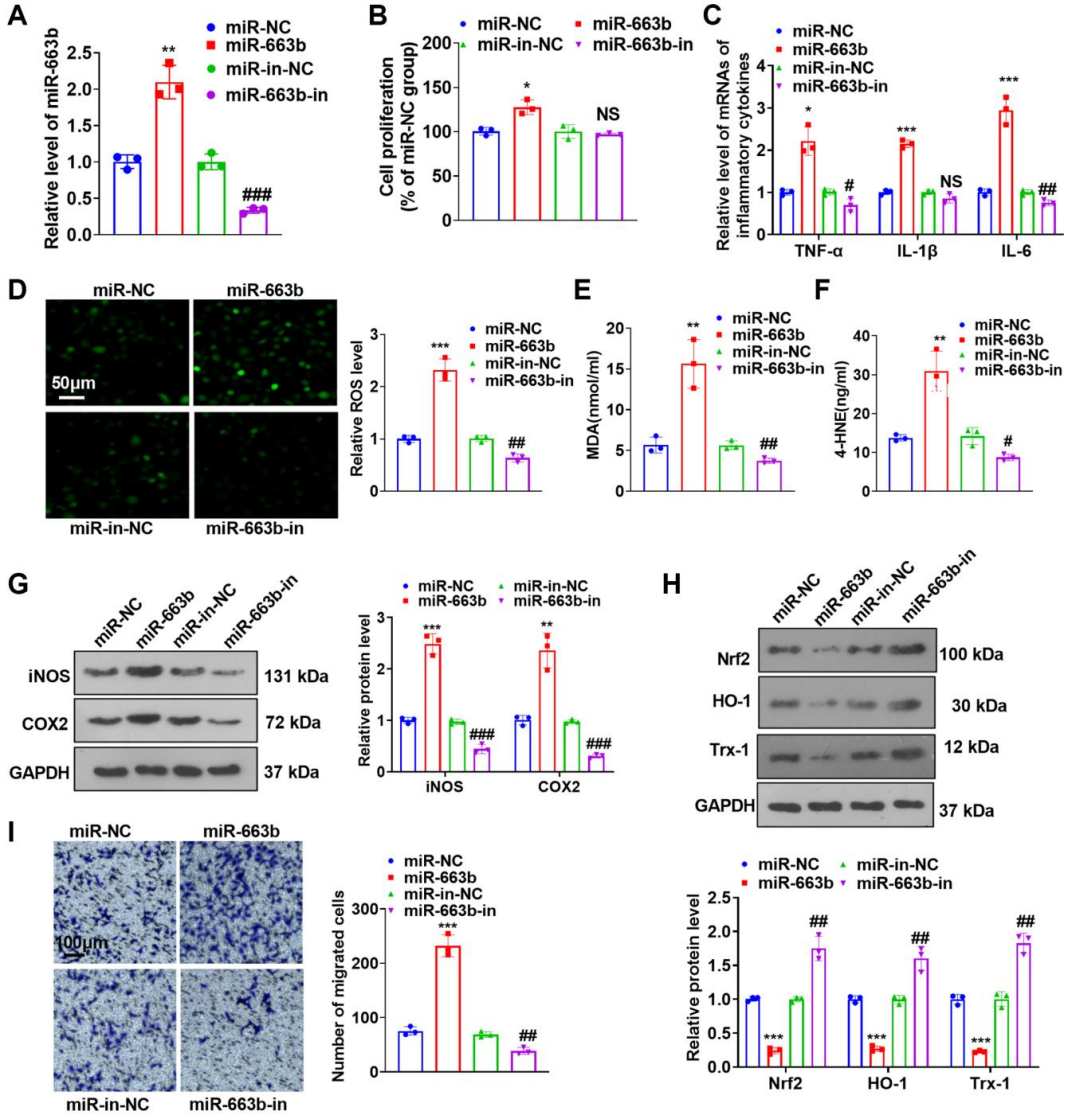
**miR-663b overexpression facilitated PASMC dysfunctions.** (**A**) PASMCs were transfected along with miR-NC, miR-663b mimics, miR-in, and miR-663b inhibitors, with RT-PCR implemented 48 hours later to confirm miR-663b expression in the transfected cells. (**B**) CCK8 assay was utilized for examining cell proliferation. (**C**) RT-PCR checked the levels of inflammatory cytokines TNF-α, IL-1β, and IL-6 in PASMCs. (**D**–**F**) Cell immunofluorescence and colorimetry determined the levels of ROS (**D**), MDA (**E**), and 4-HNE (**F**) in PASMCs. (**G**, **H**) Western blot revealed the profiles of inflammation-concerned proteins iNOS and COX2 and oxidative stress-correlated proteins Nrf2, HO-1 and Trx-1 in PASMCs. (**I**) Transwell monitored PASMC migration. *N* = 3. ^*^P < 0.05, ^**^P < 0.01, ^***^P < 0.001 (vs. miR-NC); *NS P* > 0.05, ^#^*P* < 0.05, ^##^*P* < 0.01, ^###^*P* < 0.001 (vs. miR-in-NC).

To investigate the influence of miR-663b on PASMC dysfunction, we transfected miR-NC, miR-663b mimics, miR-in-NC, and miR-663b inhibitors into PASMCs and implemented RT-PCR to verify the transfection efficiency (Figure 2A). CCK8 assay reflected that miR-663b overexpression facilitated PASMC proliferation (Figure 2B). The data of RT-PCR reflected that miR-663b enhancement led to increased profiles of inflammatory factors (TNF-α, IL-1β, and IL-6), whereas miR-663b down-expression reduced the levels of the inflammatory factors in the cells (Figure 2C). Cell immunofluorescence and colorimetry displayed that miR-663b overexpression aggravated the production of ROS levels and MDA and 4-HNE contents in PASMCs. miR-663b inhibition showed reversed results (Figure 2D–2F). Western blot indicated that PASMCs subsequent to miR-663b overexpression had an uplift in the profiles of inflammation-concerned proteins iNOS and COX2 and a decrease in those of oxidative stress-correlated proteins Nrf2, HO-1, and Trx-1. By contrast, miR-663b low expression resulted in the opposite phenomena (Figure 2G, 2H). As evidenced by Transwell assay, miR-663b overexpression fortified PASMC migration and miR-663b low expression reduced cell migration (Figure 2I). All the data revealed that miR-663b overexpression aggravated the proliferation, inflammation, oxidative stress, and migration of PASMCs.

### miR-663b overexpression boosted hypoxia-elicited PASMC dysfunction

**Figure 3:**
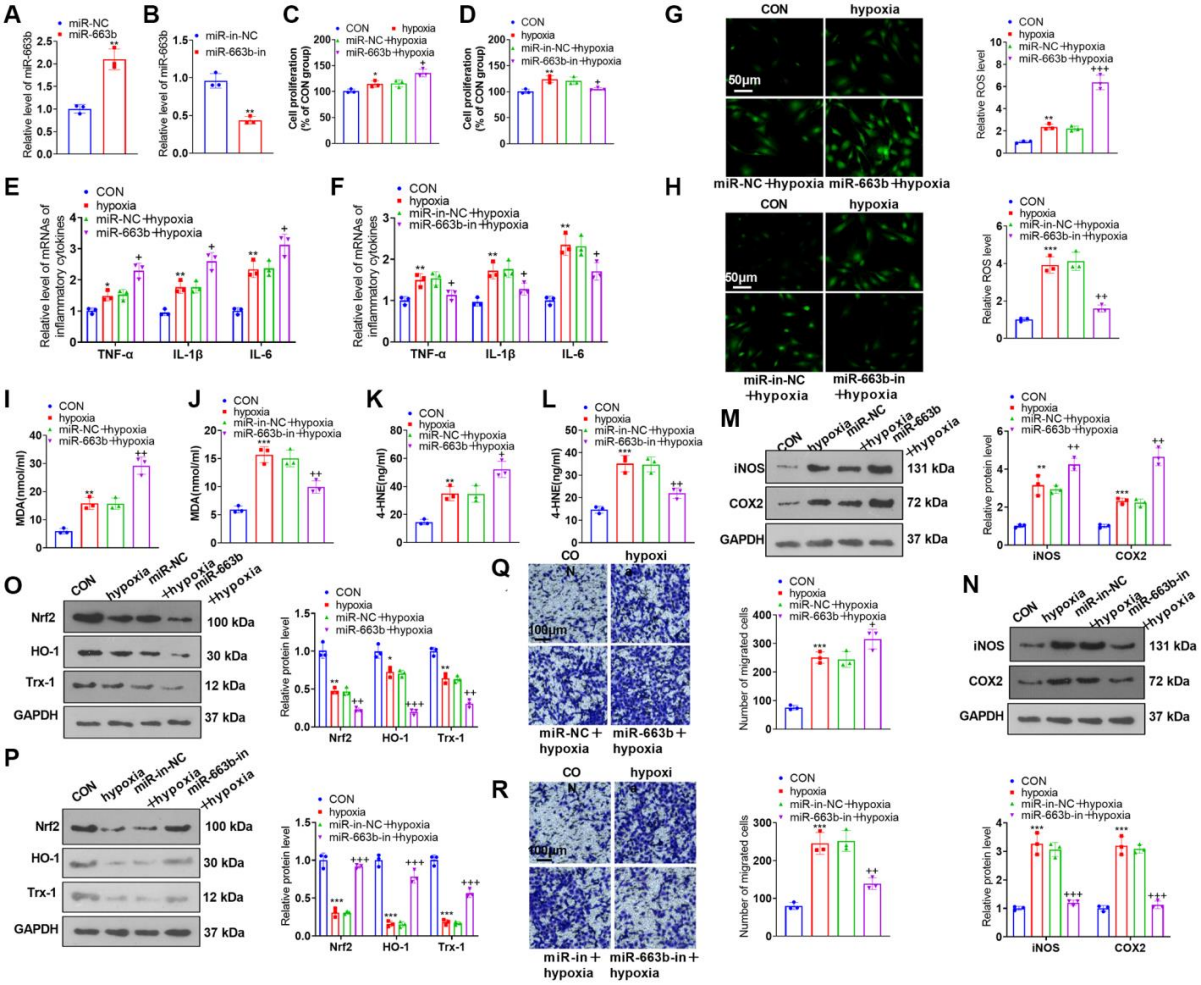
**miR-663b overexpression facilitated PASMC damage induced by hypoxia.** (**A**, **B**) PASMCs were transfected along with miR-NC, miR-663b mimics, miR-in, and miR-663b inhibitors, with RT-PCR implemented 48 hours later to confirm miR-663b expression in the transfected cells. Then, hypoxia was harnessed for 24-hour treatment of the transfected cells. (**C**, **D**) CCK8 assay was conducted for examining cell proliferation. (**E**, **F**) RT-PCR checked the levels of inflammatory cytokines TNF-α, IL-1β, and IL-6 in PASMCs. (**G**–**L**) Cell immunofluorescence and colorimetry determined the levels of ROS (**G**, **H**), MDA (**I**, **J**), and 4-HNE (**K**, **L**) in PASMCs. (**M**–**P**) Western blot was performed for assaying the profiles of inflammation-concerned proteins iNOS and COX2 and oxidative stress-correlated proteins Nrf2, HO-1 and Trx-1 in PASMCs. (**Q**, **R**) Transwell monitored PASMC migration. *N* = 3. ^*^P < 0.05, ^**^P < 0.01, ^***^P < 0.001 (vs. miR-NC, miR-in-NC, or CON); ^+^*P* < 0.05, ^++^*P* < 0.01, ^+++^*P* < 0.001 (vs. miR-NC+hypoxia or miR-in-NC+hypoxia).

To investigate the influence of miR-663b on PASMC damage induced by hypoxia, we transfected miR-NC, miR-663b mimics, miR-in, and miR-663b inhibitors into PASMCs and implemented RT-PCR to verify the transfection efficiency (Figure 3A, 3B). Functional assays showed that hypoxia strengthened PASMC proliferation, inflammatory cytokine levels, and oxidative stress (Figure 3C–3L). When contrasted with the hypoxia+miR-NC group, miR-663b overexpression bolstered cell proliferation, inflammation, and oxidative stress. However, miR-663b inhibition reversed the dysfunctions of PASMCs under hypoxia treatment (Figure 3C–3L). Western blot indicated that PASMCs subsequent to hypoxia treatment gained upregulated expression of iNOS and COX2, and downregulated Nrf2, HO-1 and Trx-1 levels. Overexpressing miR-663b enhanced iNOS and COX2 expressions and restrained Nrf2, HO-1, and Trx-1 expressions, but miR-663b low expression resulted in the opposite phenomena (Figure 3M–3P). Transwell assay showed that hypoxia promoted PASMC migration, which was further enhanced by miR-663b overexpression and decreased by miR-663b inhibition (Figure 3Q, 3R). All our outcomes revealed that miR-663b overexpression stepped up proliferation, inflammation, oxidative stress, and migration in hypoxia-induced PASMCs, but miR-663b inhibition reversed the situation.

### miR-663b presented a high profile in M1 macrophage exosomes, and M1 macrophage exosomes elicited PASMC dysfunctions

**Figure 4:**
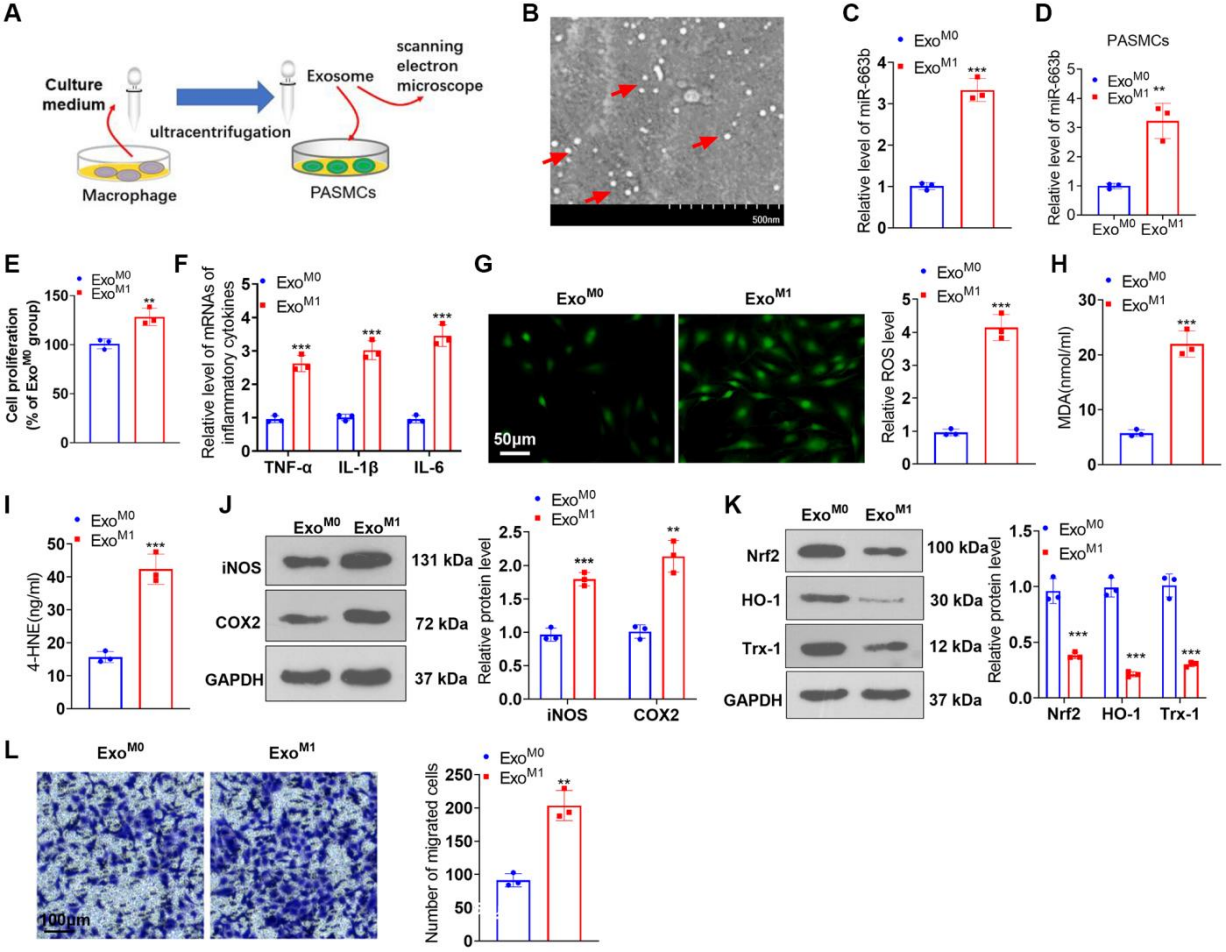
**miR-663b presented a high level in M1 macrophage exosomes, and M1 macrophage exosomes elicited PASMC damage.** (**A**) The exosomes of M0 and M1 macrophages were acquired through ultracentrifugation. (**B**) The isolated exosomes were identified by scanning electron microscope (SEM). Scale bar = 500 nm. (**C**) RT-PCR was carried out to check miR-663b expression in M0 and M1 macrophage exosomes. M1 macrophage exosomes were cultured together with PASMCs. (**D**) RT-PCR was carried out to check miR-663b expression in PASMCs. (**E**) CCK8 assay was conducted for examining PASMC proliferation. (**F**) RT-PCR checked the profiles of inflammatory factors in PASMCs. (**G**–**I**) Cell immunofluorescence and colorimetry determined the levels of ROS, MDA, and 4-HNE in PASMCs. (**J**, **K**) Western blot confirmed the profiles of inflammation-concerned proteins and oxidative stress-associated proteins in PASMCs. (**L**) Transwell measured PASMC migration. *N* = 3. ^*^P < 0.05, ^**^P < 0.01, ^***^P < 0.001 (vs. Exo^M0^).

To understand the impact of M1 macrophages on PASMCs, we constructed a co-culture model of M1 macrophages and PASMCs (Supplementary Figure 1A). The functional assays showed that M1-polarized macrophages induced enhanced PASMC proliferation, inflammation, oxidative stress, and migration (Supplementary Figure 1B–1I). We harvested “M0” and “M1” macrophage exosomes through ultracentrifugation (Figure 4A). Scanning electron microscope (SEM) showed that the diameter of “exosomes” mainly ranges from 40–100 nm (Figure 4B). RT-PCR demonstrated that in contrast with the Exo^M0^ group, miR-663b expression was substantially up-regulated in M1 macrophage exosomes (Figure 4C). To probe the influence of M1 macrophage exosomes on PASMCs, we cultured PASMCs together with M0 and M1 macrophage exosomes (Exo^M0^, Exo^M1^). MiR-663b level was upregulated in PASMCs under Exo^M1^ treatment (Figure 4D). CCK8 assay indicated that by contrast to the Exo^M0^ group, Exo^M1^ boosted PASMC proliferation (Figure 4E). RT-PCR revealed that as opposed to the Exo^M0^ group, the Exo^M1^ group had a distinct increase in the profiles of inflammatory factors in PASMCs (Figure 4F). Cell immunofluorescence and colorimetry reflected that in contrast with the Exo^M0^ group, M1 macrophage exosomes elicited an increase in the levels of ROS, MDA, and 4-HNE in PASMCs (Figure 4G–4I). Western blot indicated that by contrast to the Exo^M0^ group, iNOS and COX2 expressions were considerably up-regulated, while Nrf2, HO-1, and Trx-1 expressions were vigorously down-regulated in PASMCs in the Exo^M1^ group (Figure 4J, 4K). Transwell suggested that in contrast with the Exo^M0^ group, M1 macrophage exosomes bolstered PASMC migration (Figure 4L). These findings confirmed that miR-663b was enriched in M1 macrophage exosomes, and M1 macrophage exosomes induced PASMC damage.

### miR-663b targeted AMPK

**Figure 5:**
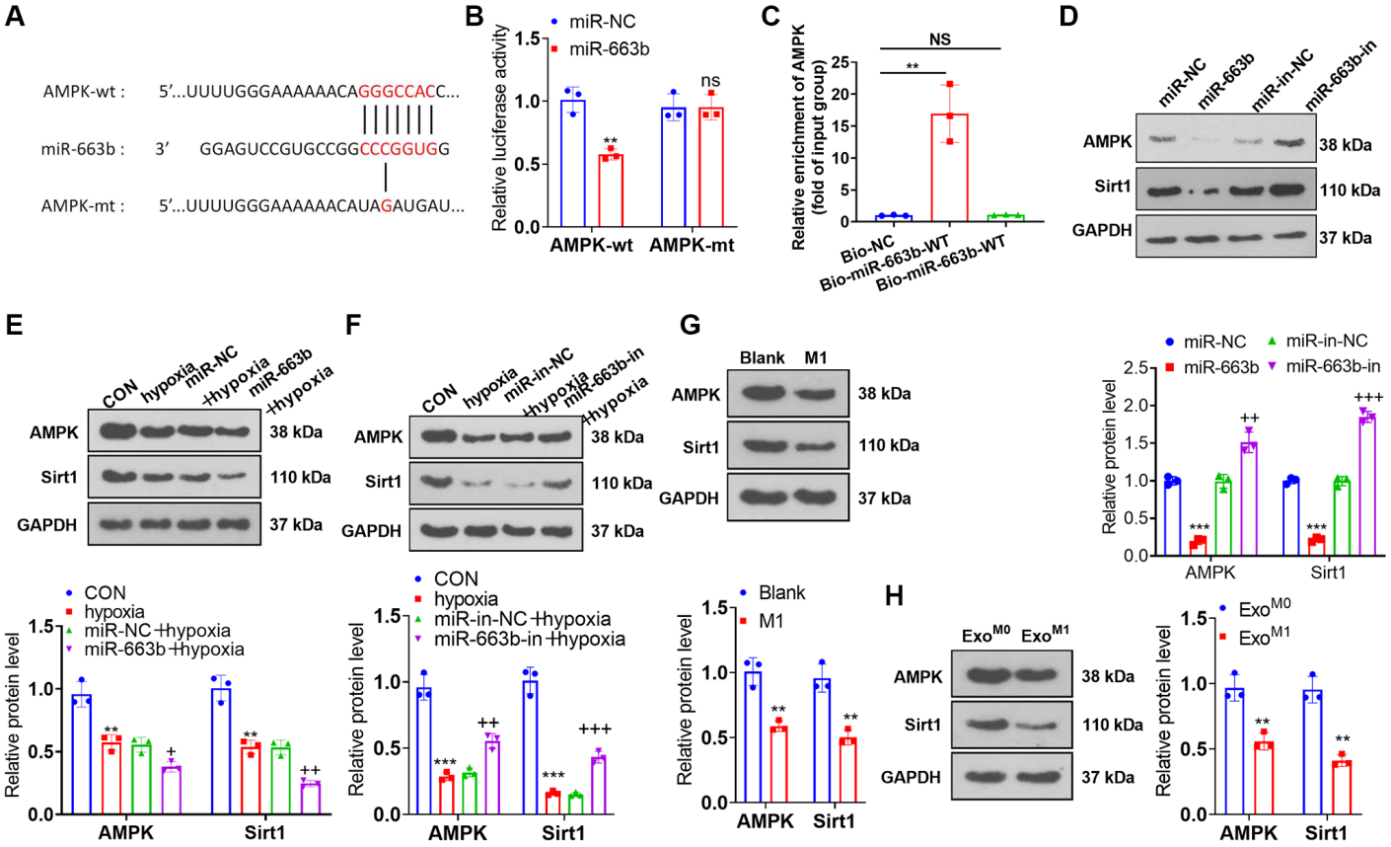
**miR-663b targeted AMPK.** (**A**, **B**) With the wild type (wt) and mutant type (mt) sequences of AMPK mRNA 3′-UTR built, dual luciferase activity assay was implemented to check the luciferase activity of 293T cells transfected along with miR-663b mimics, miR-NC, and wt or mt-AMPK 3′-UTR. (**C**) Verification of the relationship between miR-663b and AMPK by RNA pull-down assay. (**D**–**H**) Western blot confirmed the profile of the AMPK and Sirt1 in PASMCs. *N* = 3. ns *P* > 0.05, ^**^P < 0.01, ^***^P < 0.001 (vs. miR-NC, Blank, or Exo^M0^); ^+^*P* < 0.05, ^++^*P* < 0.01, ^+++^*P* < 0.001 (vs. miR-NC+hypoxia or miR-in+hypoxia).

Through Targetscan database (https://www.targetscan.org/vert_72/) and microT databse (https://dianalab.e-ce.uth.gr/html/dianauniverse/index.php?r=microT_CDS), we found 288 gene targets are potentially targeted by miR-663b (Supplementary Figure 2A). The miRNA-gene target network was shown in Supplementary Figure 2B. Moreover, gene enrichment analysis was performed through the online database DAVID (https://david.ncifcrf.gov/home.jsp). The enriched KEGG pathways and biological themes, particularly GO terms (including biological process (BP), cellular component (CC), and molecular function (MF)), were shown in Supplementary Figure 2C–2F. Dual luciferase activity assay revealed miR-663b mimics vigorously attenuated the luciferase activity of AMPK-wt (versus miR-NC) (Figure 5A, 5B). RNA pull-down analysis showed that endogenous AMPK mRNA level was enriched specifically in miR-663b probe detection compared with Bio-NC group (Figure 5C). Western blot indicated that the profiles of AMPK and Sirt1 were lowered in PASMCs with miR-663b upregulated or treated with hypoxia. In contrast with the corresponding control group, miR-663b overexpression dampened AMPK and Sirt1 expressions, whereas miR-663b boosted their expressions in PASMCs elicited by hypoxia (Figure 5D–5F). In addition, the condition medium or exosomes from M1 macrophages could both repress AMPK and Sirt1 expressions in PASMCs (Figure 5G, 5H). These discoveries denoted that miR-663b targeted AMPK, and miR-663b overexpression hindered the AMPK/Sirt1 pathway.

### AMPK activation ameliorated the impacts of miR-663b overexpression on PASMCs

**Figure 6:**
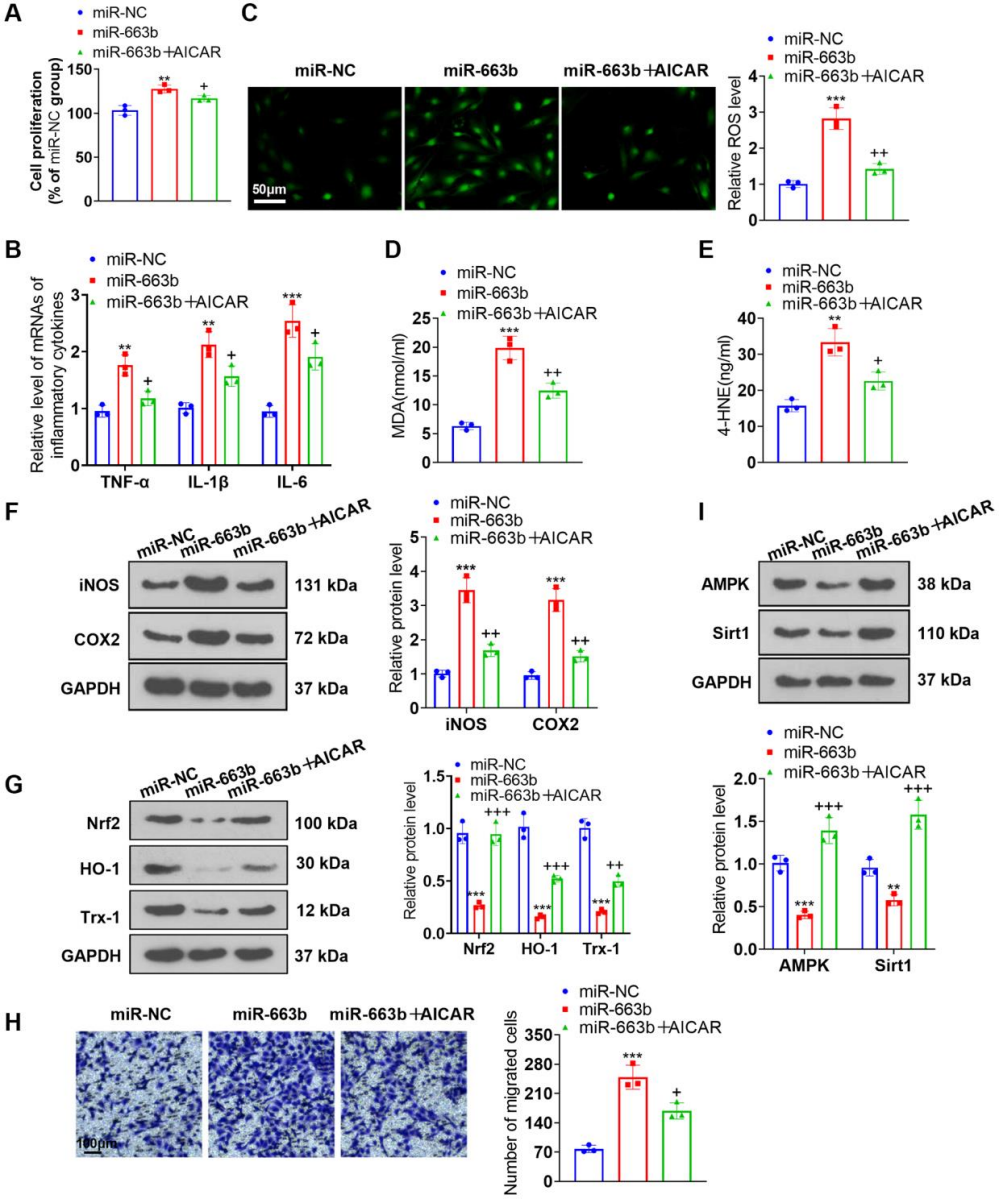
**AMPK activation alleviated the effects of miR-663b overexpression on PASMCs.** miR-663b mimics were transfected into PASMCs, with the AMPK activator AICAR applied for treatment. (**A**) CCK8 assay was conducted for examining cell proliferation. (**B**) RT-PCR measured TNF-α, IL-1β, and IL-6 profiles in PASMCs. (**C**) Cell immunofluorescence determined ROS levels in PASMCs. (**D**, **E**) Colorimetry confirmed MDA and 4-HNE contents in PASMCs. (**F**) Western blot verified iNOS and COX2 levels in PASMCs. (**G**) Western blot checked Nrf2, HO-1 and Trx-1 expressions in PASMCs. (**H**) Transwell monitored PASMC migration. (**I**) Western blot figured out the profiles of AMPK and Sirt1 in PASMCs. *N* = 3. ^**^P < 0.01, ^***^P < 0.001 (vs. miR-NC); ^+^*P* < 0.05, ^++^*P* < 0.01, ^+++^*P* < 0.001 (vs. miR-663b).

To ascertain the function of AMPK in PASMC damage, we transfected miR-663b mimics into PASMCs and applied the AMPK activator AICAR. As supported by functional assays, miR-663b overexpression boosted PASMC proliferation, the levels of inflammatory cytokines, ROS, MDA, and 4-HNE, but when contrasted to the miR-663b group, the group of miR-663b+AICAR underwent a reduction in cell proliferation, the levels of inflammatory cytokines, ROS levels and in MDA and 4-HNE contents (Figure 6A–6E). Western blot unveiled that iNOS and COX2 expressions went up, while Nrf2 and Trx-1 expressions were down in the miR-663b group vis-a-vis the miR-NC group. In contrast with the miR-663b group, AICAR restrained the profiles of iNOS and COX2 but enhanced those of Nrf2, HO-1, and Trx-1 (Figure 6F, 6G). Transwell reflected that vis-à-vis the miR-NC group, overexpressing miR-663b stepped up PASMC migration, but it was impeded following the administration of AICAR (Figure 6H). Western blot revealed that versus the miR-NC group, overexpressing miR-663b cramped AMPK and Sirt1 expressions, but they were heightened by AICAR (Figure 6I). These findings confirmed that AMPK activation mitigated the influence of miR-663b overexpression on PASMCs.

### AMPK activation alleviated the impact of M1 macrophage exosomes on PASMCs dysfunction

**Figure 7:**
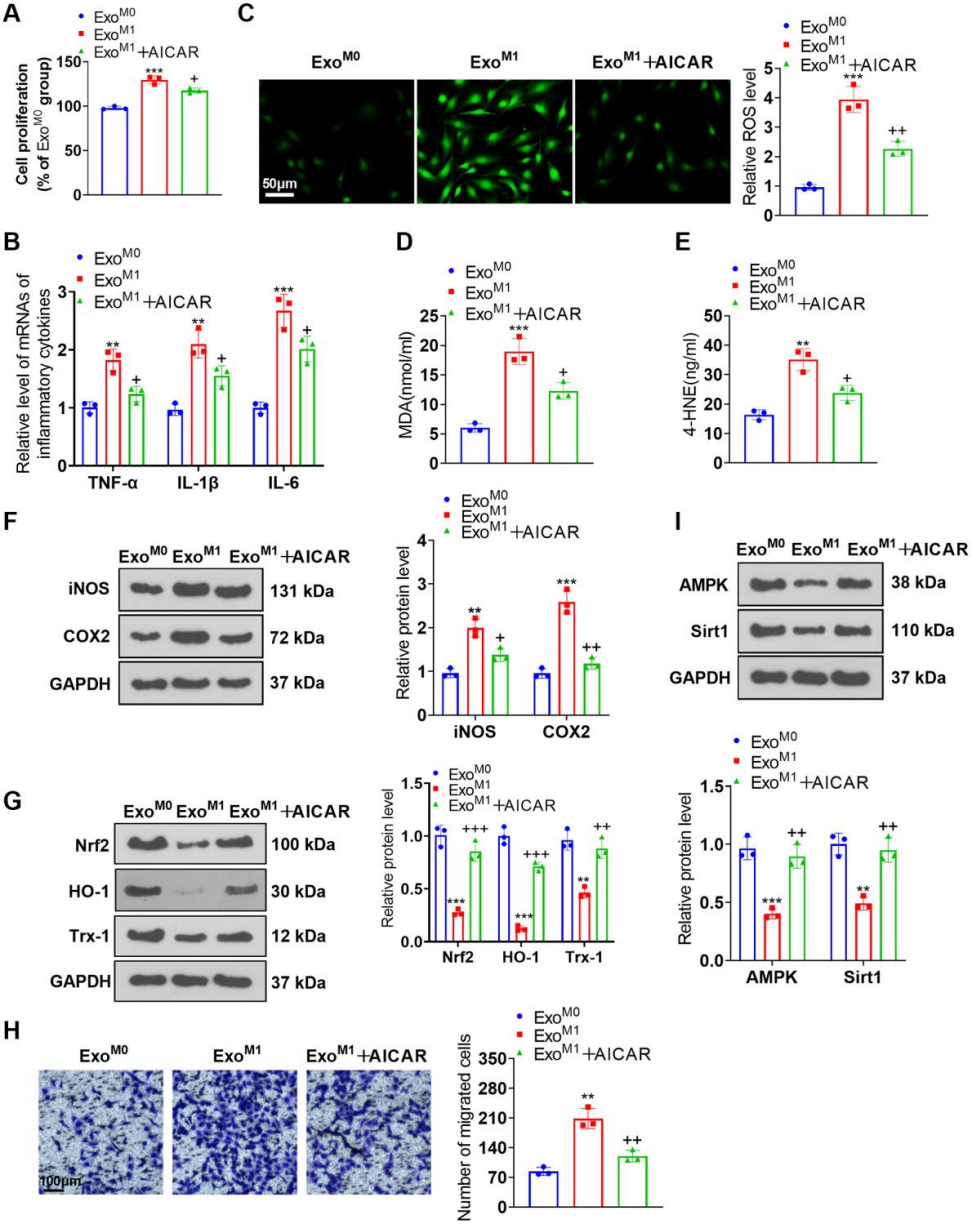
**AMPK activation ameliorated the impacts of M1 macrophage exosomes on PASMCs.** PASMCs were cultured together with M0 and M1 macrophage exosomes, with AICAR applied to the conditioned medium. (**A**) CCK8 assay was conducted for examining PASMC proliferation. (**B**) RT-PCR was taken for gauging TNF-α, IL-1β, and IL-6 expressions in PASMCs. (**C**–**E**) Cell immunofluorescence and colorimetry confirmed the levels of ROS, MDA, and 4-HNE in PASMCs. (**F**, **G**) Western blot was done for measuring iNOS, COX2, Nrf2, HO-1 and Trx-1 expressions in PASMCs. (**H**) Transwell examined PASMC migration. (**I**) Western blot verified the level of the AMPK/Sirt1 pathway in PASMCs. *N* = 3. ^**^P < 0.01, ^***^P < 0.001 (vs. Exo^M0^); ^+^*P* < 0.05, ^++^*P* < 0.01, ^+++^*P* < 0.001 (vs. Exo^M1^).

We cultured PASMCs together with the exosomes of M0 and M1 macrophages and then applied the AMPK activator AICAR for treatment. AICAR suppressed PASMC proliferation (Figure 7A), the profiles of inflammatory factors (Figure 7B), the levels of ROS, MDA, and 4-HNE (Figure 7C–7E). Western blot unraveled that iNOS and COX2 expressions were abated, while Nrf2, HO-1, and Trx-1 expressions were elevated in the Exo^M1^+AICAR group vis-a-vis the Exo^M1^ group (Figure 7F, 7G). Transwell denoted that in contrast with the Exo^M1^ group, AICAR vigorously dampened PASMC migration (Figure 7H). Western blot disclosed that there was a reduction in AMPK and Sirt1 expressions in the Exo^M1^ group versus the Exo^M0^ group. In contrast with the Exo^M1^ group, AICAR boosted AMPK and Sirt1 expressions in PASMCs (Figure 7I). These phenomena demonstrated that AMPK activation mitigated the damaging impact of M1 macrophage exosomes on PASMCs.

### M1 macrophage exosomes with miR-663b low expression ameliorated pulmonary vascular remodeling in rats suffering from pulmonary hypertension

**Figure 8:**
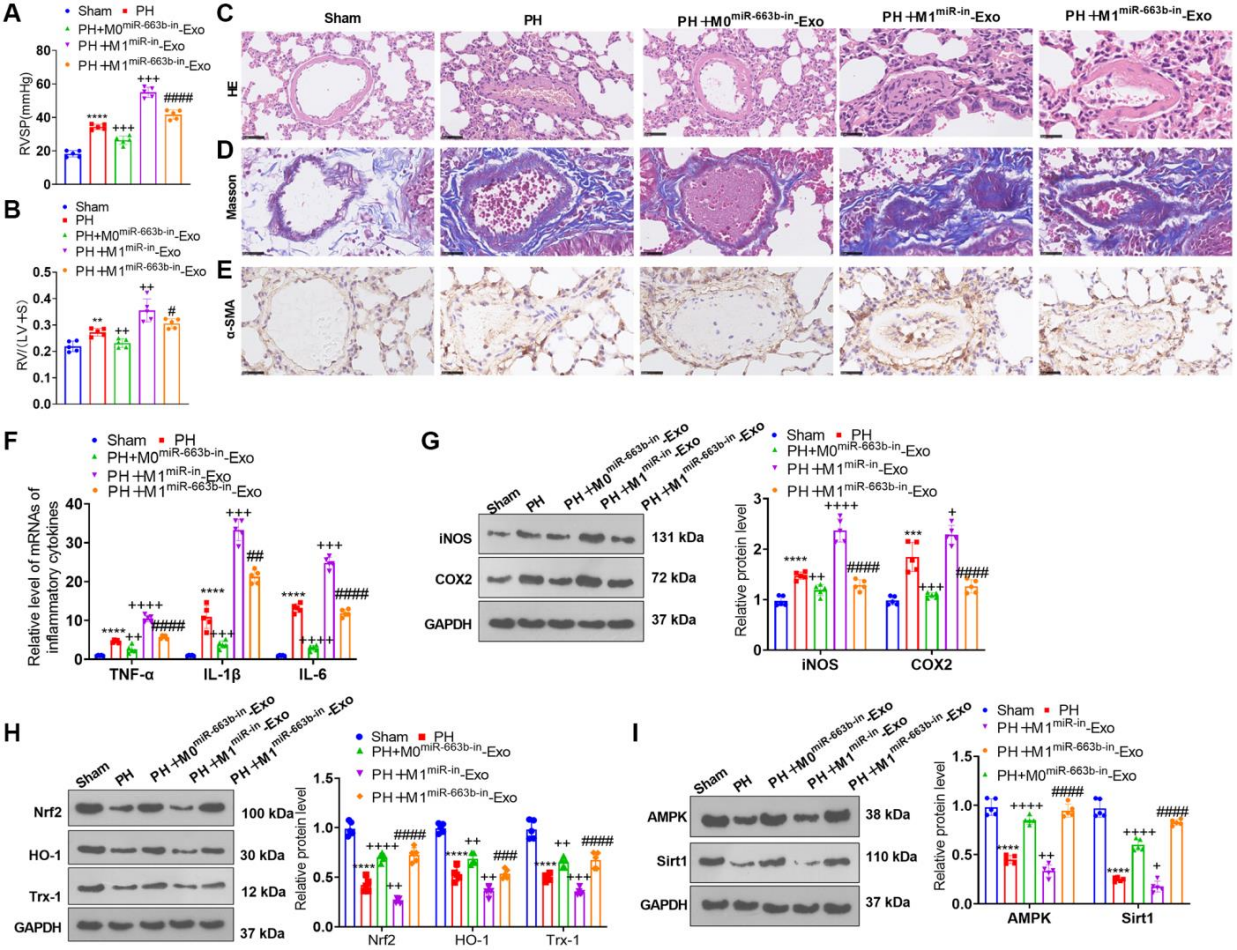
**M1 macrophage exosomes with miR-663b low expression ameliorated pulmonary vascular remodeling in pulmonary hypertension rats.** A pulmonary hypertension rat model elicited by hypoxia was built, and M1 macrophage exosomes with low-expressed miR-663b were transfused into the caudal veins of the rats. (**A**, **B**) The hemodynamic assay was carried out to confirm the mean ratio of RVSP and RV/(LV+S) of PH rats. (**C**, **D**) HE staining (**C**) and Masson staining (**D**) were conducted for monitoring pathological alterations in the lung tissues of PH rats. (**E**) Immunohistochemistry (anti-α-SMA) was performed for detecting pulmonary vascular remodeling. (**F**) RT-PCR was implemented for gauging TNF-α, IL-1β, and IL-6 levels in the tissues. (**G**, **H**) iNOS, COX2, Nrf2, HO-1 and Trx-1 profiles in the tissues were confirmed through western blot. (**I**) The level of the AMPK/Sirt1 pathway in the tissues was ascertained by western blot. *N* = 5. ^**^P < 0.01, ^***^P < 0.001, ^****^P < 0.0001 (vs. Sham); ^++++^*P* < 0.0001 (vs. PH); ^####^*P* < 0.0001 (vs. PH+M1^miR-in^-Exo).

We built a pulmonary hypertension rat model induced by hypoxia and transfused M1 macrophage exosomes with low-expressed miR-663b into the caudal veins of the rats with a view to further corroborating the function of miR-663b in pulmonary hypertension. The hemodynamic assay revealed that the mean ratio of RVSP and RV/(LV+S) was remarkably higher in the PH group than in the Sham group. As compared with the PH group, the PH+M0^miR-663b-in^-Exo group had reduced RVSP and RV/(LV+S), and the PH+M1^miR-in^-Exo witnessed a notable increase in the mean ratio of RVSP and RV/(LV+S). In contrast with the PH+M1^miR-in^-Exo group, M1 macrophage exosomes with low-expressed miR-663b contributed to a stark reduction in the ratio (Figure 8A, 8B). HE staining, Masson staining, and IHC displayed that by contrast to the Sham group, PH rats manifested a reduced inner diameter of the pulmonary arteriole, narrowed lumen, and significant pulmonary remodeling. In contrast with the PH group, the rats in the PH+M1^miR-in^-Exo group exhibited thickened pulmonary arterioles, but the PH+M0^miR-663b-in^-Exo group and M1 macrophage exosomes with low-expressed miR-663b brought about a decrease in the thickness of the PH rat pulmonary artery media and distinctly alleviated the pathological damage of the lung tissues (Figure 8C–8E). RT-PCR denoted that by contrast to the Sham group, there was an increase in the levels of TNF-α, IL-1β, and IL-6 in the PH rat lung tissues. As opposed to the PH group, the PH+M1^miR-in^-Exo group displayed a dramatic uplift in the profiles of the inflammatory factors, while the PH+M0^miR-663b-in^-Exo group had a reduced inflammatory response. By contrast to the PH+M1^miR-in^-Exo group, M1 macrophage exosomes with miR-663b low expression lowered the profiles of the inflammatory factors in the PH rat lung tissues (Figure 8F). As evidenced by western blot, the profiles of iNOS and COX2 were heightened, and Nrf2, HO-1, and Trx-1 expressions were diminished in the lung tissues of the PH rats. The PH+M0^miR-663b-in^-Exo group had reduced profiles of inflammation-concerned proteins. Moreover, iNOS and COX2 proteins were dramatically elevated, and Nrf2, HO-1, and Trx-1 proteins were conspicuously abated in the PH+M1^miR-in^-Exo group vis-a-vis the PH group. By contrast to the PH+M1^miR-in^-Exo group, M1 macrophage exosomes with low-expressed miR-663b suppressed the profiles of inflammation-correlated proteins and enhanced those of oxidative stress-concerned proteins in the PH rat lung tissues (Figure 8G, 8H). Western blot revealed that in contrast with the Sham group, the profiles of AMPK and Sirt1 were attenuated in the PH rat lung tissues. As opposed to the PH group, the PH+M0^miR-663b-in^-Exo group had enhanced AMPK-Sirt1 expression, and the PH+M1^miR-in^-Exo group experienced a distinct reduction in their expressions. By contrast to the PH+M1^miR-in^-Exo group, M1 macrophage exosomes with miR-663b low expression heightened the profile of the AMPK/Sirt1 pathway in the PH rats’ lung tissues (Figure 8I). All our findings confirmed that M1 macrophage exosomes with miR-663b low expression mitigated pulmonary vascular remodeling in pulmonary hypertension rats.

## DISCUSSION

**Figure 9:**
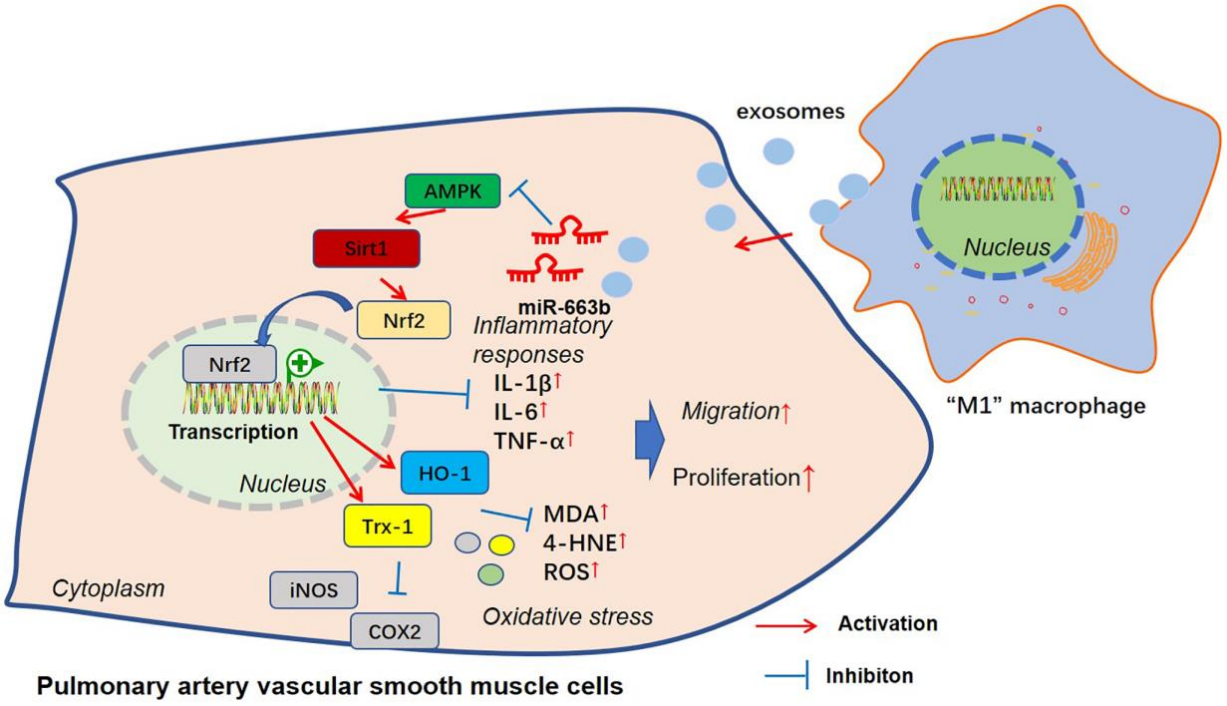
The mechanism sketch of exosomal miR-663b from “M1” macrophages in regulating PASMC dysfunction.

Pulmonary hypertension refers to a clinical and pathophysiological syndrome characterized by heightened pulmonary vascular resistance and pulmonary artery pressure resulting from structural or functional alterations in pulmonary vessels due to miscellaneous pathogenic mechanisms, thus progressing into right heart failure or even death [25]. Exosomes, carrying specific proteins, mRNAs, miRNAs, and other contents, partake in physiological processes like cell communication and migration, pertaining to the occurrence and progression of umpteen diseases [26]. Exosome-derived miRNAs have extensive participation in the occurrence and progression of cardiovascular diseases like heart failure, myocardial ischemia-reperfusion damage, and pulmonary hypertension [27]. Here, we discovered that exosomal miR-663b from M1-macrophage induces multifunction disorders of PASMCs through dampening the AMPK/Sirt1 pathway activation (Figure 9).

miRNAs function as essential mediators in homeostasis and pathological alterations of diseases [28]. Recently, many miRNAs have been found to induce abnormal functions of pulmonary artery smooth muscle cells and get involved in pulmonary vascular remodeling [29–31]. For instance, miR-150 has been shown to play a significant role in regulating pulmonary artery smooth muscle cells (PASMCs) and pulmonary artery endothelial cells (PAECs) phenotypes and functions and also affects hypoxia-induced pulmonary vascular remodeling and fibrosis [32]. miR-663 has been shown to play a role in various cellular processes, such as cell proliferation, differentiation, and apoptosis [33]. Studies have found that miR-663 is dysregulated in various types of cancers [34–35]. We performed experiments for measuring the functions of miR-663b in PASMC. Our data showed that miR-663b overexpression also aggravates the dysfunctions of PASMC. M1 macrophages, referred to as classical macrophages, can generate pro-inflammatory cytokines, mainly playing a role in facilitating inflammation occurrence and development, sterilization, and phagocytosis [36]. Pulmonary inflammation mediated by macrophages surrounding pulmonary vessels is a critical driving factor for pulmonary vascular remodeling. Macrophages, in different microenvironments, can be polarized into M1 or M2 type, hence influencing pulmonary hypertension progression [37]. In the PAH rat model elicited by monocrotaline (MCT), M1 macrophages can step up endothelial cell apoptosis to take part in the early stage of inflammation, and M2 macrophages can boost endothelial cell and smooth muscle proliferation to participate in inflammation repair and the following remodeling stage of abnormal tissues [38]. Over the past years, extracellular vesicles originating from macrophages have become essential mediators in the pathology of many diseases like inflammatory diseases, fibrosis, and cancer [39]. In the ovalbumin (OVA)-elicited asthmatic rat model, M2 macrophage exosome miR-370 lowers FGF1 expression and down-regulates the MAPK/STAT1 signaling pathway to alleviate lung tissue fibrosis and inflammation, thus ameliorating asthma [40]. Macrophage exosome miR-142-3p attenuates the profiles of transforming growth factor β receptor 1 (TGFβ-R1) and pro-fibrosis genes to fight idiopathic pulmonary fibrosis (IPF) progression [41]. miR-663b inhibition enhances the profile of B-cell lymphoma 2 like 1 (BCL2L1) to lessen myocardial cell apoptosis and inflammation, hence defending myocardial cells from hypoxia-caused damage [42]. Our research disclosed that miR-663b was notably up-regulated in M1 macrophages and their exosomes as well as in the *in-vivo* and *in-vitro* pulmonary hypertension models. miR-663b overexpression stepped up hypoxia-elicited PASMC damage, whereas miR-663b low expression culminated in the opposite landscape. In the co-culture model, M1 macrophages and their exosomes substantially bolstered PASMC proliferation, inflammation, oxidative stress, and migration. In the animal trials, M1 macrophage exosomes suppressing miR-663b mitigated pulmonary vascular remodeling in rats suffering from pulmonary hypertension. Our findings confirmed that M1 macrophage exosome miR-663b could foster pulmonary hypertension progression.

AMPK, a critical enzyme modulating cellular energy homeostasis, takes part in hypoxic pulmonary hypertension formation and pulmonary vascular remodeling [43]. For instance, PASMCs dealing with nitric oxide (NO) gained increased expression of PDE3A, PDE3 activity, and AMPK phosphorylation. The siRNA targeting PDE3A suppressed NO-induced AMPK activation, suggesting that the potential PDE3A-AMPK axis is involved in pulmonary hypertensive disorders [44]. Many abnormal phenotypes of PASMC are found in PAH, such as increased proliferation, resistance to apoptosis, migration, oxidative stress, and inflammatory responses [45–47]. Interestingly, AMPK acts as a multifunctional mediator in those processes [48–49]. Increased mitochondrial reactive oxygen species (mROS) and glycolysis of PASMCs are identified as salient features in PAH progression [50]. The metalloenzyme glycolytic enzyme α-enolase (ENO1) mitigates PASMC proliferation and prevents the hypoxia-induced metabolic shift from mitochondrial respiration to glycolysis in PASMC. Mechanistically, ENO1 affects the abnormal phenotypes of PASMC dependently through the AMPK-Akt pathway [51]. Moreover, several AMPK activators, such as metformin [52] and Salidroside [53], also play a role in mediating the disorders of PASMC or PAH development. For instance, a recent clinical study reveals that metformin improved the right ventricular fractional area of PAH patients probably by altering lipid and glucose metabolism [54]. Under the administration of Salidroside, the altered proliferation and apoptosis resistance of hypoxia-induced pulmonary arterial smooth muscle cells were significantly improved. The underlying mechanism study revealed that Salidroside achieved its effects by promoting autophagy and activating the AMPK-mTOR-ULK1 pathway [55]. Those studies all suggest that the AMPK pathway has a potent function in mediating PASMC functions.

Several downstream proteins are affected by AMPK mediators. As a vital transcription factor that controls inflammatory responses, NF-κB is activated during vascular disorders [56]. AMPK pathway activation represses NF-κB p65 phosphorylation and nuclear translocation, thus restraining NF-κB-mediated inflammatory reactions [57–58]. In rat models of PAH, AMPK activation by metformin represses NF-κB activation to impede autophagy and vascular remodeling, hence stopping PAH development [59]. Sirt1 is a deacetylase. Acetylation and deacetylation balance owing to Sirt1 inactivation alters pulmonary hypertension pathogenesis [60]. When it comes to pulmonary hypertension, Sirt1 up-regulation initiates the Akt signaling pathway to facilitate hypoxia-elicited proliferation in human pulmonary artery endothelial cells and curb their apoptosis [61]. Moreover, Sirt1 activators can also curb NF-κB-mediated inflammatory reactions, thus exhibiting regulatory functions in VSMC disorders [62–63]. Notably, AMPK can initiate Sirt1, and Sirt1 in turn can activate AMPK via phosphorylation [64], this indicates that AMPK and Sirt1 interact with each other. On the other hand, activating the AMPK/Sirt1 and Nrf2/HO-1 signaling pathways alleviates oxidative stress and inflammation in lipopolysaccharide (LPS)-treated bronchial epithelioid cells [65]. Nrf2 is a transcription factor that regulates genes that contain antioxidant response elements (ARE) in their promoters [66]. Nrf2 nuclear accumulation enhances the expressions of its encoded proteins, including HO-1 and Trx-1, which are in charge of the production of free radicals and are involved in response to oxidative injury [67–68]. Nrf2 is known to regulate the expression of numerous antioxidant and detoxifying enzymes, such as glutathione peroxidase (GPx), catalase (CAT), and HO-1, which act to reduce ROS levels and restore redox homeostasis in PASMC functions and pulmonary vascular remodeling [69–71].

Here, dual luciferase activity assay substantiated the targeted association between miR-663b and AMPK. As for the mechanism, miR-663b overexpression restrained the Nrf2-HO-1 and Trx-1 and AMPK/Sirt1 pathways, while AMPK activation lessened the damaging impact of miR-663b overexpression and M1 macrophage exosomes on PASMCs. All these phenomena revealed that miR-663b modulated the AMPK/Sirt1 signaling pathway to exert a function in pulmonary hypertension development.

All in all, we have corroborated that miR-663b, substantially enriched in M1 macrophage exosomes, suppresses the AMPK/Sirt1 pathway to bolster PASMC proliferation, inflammation, oxidative stress, and migration (Figure 9). Our research outcomes may well provide new targets for pulmonary hypertension prevention and treatment.

## Supplementary Materials

Supplementary Figures

## References

[1] Poch D, Mandel J. Pulmonary Hypertension. Ann Intern Med. 2021; 174:ITC49–64. 10.7326/AITC202104200 33844574

[2] Crosswhite P, Sun Z. Nitric oxide, oxidative stress and inflammation in pulmonary arterial hypertension. J Hypertens. 2010; 28:201–12. 10.1097/HJH.0b013e328332bcdb 20051913 PMC2809140

[3] Shimoda LA, Laurie SS. Vascular remodeling in pulmonary hypertension. J Mol Med (Berl). 2013; 91:297–309. 10.1007/s00109-013-0998-0 23334338 PMC3584237

[4] Zahid KR, Raza U, Chen J, Raj UJ, Gou D. Pathobiology of pulmonary artery hypertension: role of long non-coding RNAs. Cardiovasc Res. 2020; 116:1937–47. 10.1093/cvr/cvaa050 32109276

[5] Draijer C, Robbe P, Boorsma CE, Hylkema MN, Melgert BN. Dual role of YM1+ M2 macrophages in allergic lung inflammation. Sci Rep. 2018; 8:5105. 10.1038/s41598-018-23269-7 29572536 PMC5865212

[6] Al-Qazazi R, Lima PDA, Prisco SZ, Potus F, Dasgupta A, Chen KH, Tian L, Bentley RET, Mewburn J, Martin AY, Wu D, Jones O, Maurice DH, et al. Macrophage-NLRP3 Activation Promotes Right Ventricle Failure in Pulmonary Arterial Hypertension. Am J Respir Crit Care Med. 2022; 206:608–24. 10.1164/rccm.202110-2274OC 35699679 PMC9716901

[7] Abid S, Marcos E, Parpaleix A, Amsellem V, Breau M, Houssaini A, Vienney N, Lefevre M, Derumeaux G, Evans S, Hubeau C, Delcroix M, Quarck R, et al. CCR2/CCR5-mediated macrophage-smooth muscle cell crosstalk in pulmonary hypertension. Eur Respir J. 2019; 54:1802308. 10.1183/13993003.02308-2018 31320454

[8] Wang C, Ma C, Gong L, Guo Y, Fu K, Zhang Y, Zhou H, Li Y. Macrophage Polarization and Its Role in Liver Disease. Front Immunol. 2021; 12:803037. 10.3389/fimmu.2021.803037 34970275 PMC8712501

[9] Zawia A, Arnold ND, West L, Pickworth JA, Turton H, Iremonger J, Braithwaite AT, Cañedo J, Johnston SA, Thompson AAR, Miller G, Lawrie A. Altered Macrophage Polarization Induces Experimental Pulmonary Hypertension and Is Observed in Patients With Pulmonary Arterial Hypertension. Arterioscler Thromb Vasc Biol. 2021; 41:430–45. 10.1161/ATVBAHA.120.314639 33147993 PMC7752239

[10] Lai X, Zhong J, Zhang B, Zhu T, Liao R. Exosomal Non-Coding RNAs: Novel Regulators of Macrophage-Linked Intercellular Communication in Lung Cancer and Inflammatory Lung Diseases. Biomolecules. 2023; 13:536. 10.3390/biom13030536 36979471 PMC10046066

[11] Zeng J, Gu C, Sun Y, Chen X. Engineering of M2 Macrophages-Derived Exosomes via Click Chemistry for Spinal Cord Injury Repair. Adv Healthc Mater. 2023; e2203391. [Epub ahead of print]. 10.1002/adhm.202203391 36877863

[12] Zhang J, Li S, Li L, Li M, Guo C, Yao J, Mi S. Exosome and exosomal microRNA: trafficking, sorting, and function. Genomics Proteomics Bioinformatics. 2015; 13:17–24. 10.1016/j.gpb.2015.02.001 25724326 PMC4411500

[13] Chen J, Hu C, Pan P. Extracellular Vesicle MicroRNA Transfer in Lung Diseases. Front Physiol. 2017; 8:1028. 10.3389/fphys.2017.01028 29311962 PMC5732924

[14] Zhang S, Liu J, Zheng K, Chen L, Sun Y, Yao Z, Sun Y, Lin Y, Lin K, Yuan L. Exosomal miR-211 contributes to pulmonary hypertension via attenuating CaMK1/PPAR-γaxis. Vascul Pharmacol. 2021; 136:106820. 10.1016/j.vph.2020.106820 33238205

[15] Yang Z, Li P, Yuan Q, Wang X, Ma HH, Zhuan B. Inhibition of miR-4640-5p alleviates pulmonary hypertension in chronic obstructive pulmonary disease patients by regulating nitric oxide synthase 1. Respir Res. 2023; 24:92. 10.1186/s12931-023-02387-5 36964568 PMC10039540

[16] Peng L, Chun-guang Q, Bei-fang L, Xue-zhi D, Zi-hao W, Yun-fu L, Yan-ping D, Yang-gui L, Wei-guo L, Tian-yong H, Zhen-wen H. Clinical impact of circulating miR-133, miR-1291 and miR-663b in plasma of patients with acute myocardial infarction. Diagn Pathol. 2014; 9:89. 10.1186/1746-1596-9-89 24885383 PMC4082297

[17] You X, Sun W, Wang Y, Liu X, Wang A, Liu L, Han S, Sun Y, Zhang J, Guo L, Zhang Y. Cervical cancer-derived exosomal miR-663b promotes angiogenesis by inhibiting vinculin expression in vascular endothelial cells. Cancer Cell Int. 2021; 21:684. 10.1186/s12935-021-02379-9 34923985 PMC8684657

[18] Zhao Q, Song P, Zou MH. AMPK and Pulmonary Hypertension: Crossroads Between Vasoconstriction and Vascular Remodeling. Front Cell Dev Biol. 2021; 9:691585. 10.3389/fcell.2021.691585 34169079 PMC8217619

[19] Zhou Y, Wang Y, Wang X, Tian X, Zhang S, Yang F, Guo H, Fan R, Feng N, Jia M, Gu X, Wang Y, Li J, Pei J. The Protective Effects of Κ-Opioid Receptor Stimulation in Hypoxic Pulmonary Hypertension Involve Inhibition of Autophagy Through the AMPK-MTOR Pathway. Cell Physiol Biochem. 2017; 44:1965–79. 10.1159/000485886 29224002

[20] Rahman I, Kinnula VL, Gorbunova V, Yao H. SIRT1 as a therapeutic target in inflammaging of the pulmonary disease. Prev Med. 2012 (Suppl); 54:S20–8. 10.1016/j.ypmed.2011.11.014 22178470 PMC3311735

[21] Yu L, Tu Y, Jia X, Fang K, Liu L, Wan L, Xiang C, Wang Y, Sun X, Liu T, Yu D, Cao W, Song Y, Fan Y. Resveratrol Protects Against Pulmonary Arterial Hypertension in Rats via Activation of Silent Information Regulator 1. Cell Physiol Biochem. 2017; 42:55–67. 10.1159/000477115 28494457

[22] Yang Y, Zhong ZT, Xiao YG, Chen HB. The Activation of AMPK/NRF2 Pathway in Lung Epithelial Cells Is Involved in the Protective Effects of Kinsenoside on Lipopolysaccharide-Induced Acute Lung Injury. Oxid Med Cell Longev. 2022; 2022:3589277. 10.1155/2022/3589277 35340214 PMC8956386

[23] Li X, Jamal M, Guo P, Jin Z, Zheng F, Song X, Zhan J, Wu H. Irisin alleviates pulmonary epithelial barrier dysfunction in sepsis-induced acute lung injury via activation of AMPK/SIRT1 pathways. Biomed Pharmacother. 2019; 118:109363. 10.1016/j.biopha.2019.109363 31545277

[24] Zhuan B, Wang X, Wang MD, Li ZC, Yuan Q, Xie J, Yang Z. Hypoxia induces pulmonary artery smooth muscle dysfunction through mitochondrial fragmentation-mediated endoplasmic reticulum stress. Aging (Albany NY). 2020; 12:23684–97. 10.18632/aging.103892 33221740 PMC7762493

[25] Coons JC, Pogue K, Kolodziej AR, Hirsch GA, George MP. Pulmonary Arterial Hypertension: a Pharmacotherapeutic Update. Curr Cardiol Rep. 2019; 21:141. 10.1007/s11886-019-1235-4 31758342

[26] Xu JY, Chen GH, Yang YJ. Exosomes: A Rising Star in Falling Hearts. Front Physiol. 2017; 8:494. 10.3389/fphys.2017.00494 28751864 PMC5508217

[27] Zheng D, Huo M, Li B, Wang W, Piao H, Wang Y, Zhu Z, Li D, Wang T, Liu K. The Role of Exosomes and Exosomal MicroRNA in Cardiovascular Disease. Front Cell Dev Biol. 2021; 8:616161. 10.3389/fcell.2020.616161 33511124 PMC7835482

[28] Kuang Z, Wu J, Tan Y, Zhu G, Li J, Wu M. MicroRNA in the Diagnosis and Treatment of Doxorubicin-Induced Cardiotoxicity. Biomolecules. 2023; 13:568. 10.3390/biom13030568 36979503 PMC10046787

[29] Zhang Y, Tang S, Yang W, Du F. let-7b-5p suppresses the proliferation and migration of pulmonary artery smooth muscle cells via down-regulating IGF1. Clinics (Sao Paulo). 2022; 77:100051. 10.1016/j.clinsp.2022.100051 35636162 PMC9156868

[30] Yen TA, Huang HC, Wu ET, Chou HW, Chou HC, Chen CY, Huang SC, Chen YS, Lu F, Wu MH, Tsao PN, Wang CC. Microrna-486-5P Regulates Human Pulmonary Artery Smooth Muscle Cell Migration via Endothelin-1. Int J Mol Sci. 2022; 23:10400. 10.3390/ijms231810400 36142307 PMC9499400

[31] Niu Z, Fu M, Li Y, Ren H, Zhang X, Yao L. Osthole alleviates pulmonary vascular remodeling by modulating microRNA-22-3p mediated lipid metabolic reprogramming. Phytomedicine. 2022; 96:153840. 10.1016/j.phymed.2021.153840 34836745

[32] Li Y, Ren W, Wang X, Yu X, Cui L, Li X, Zhang X, Shi B. MicroRNA-150 relieves vascular remodeling and fibrosis in hypoxia-induced pulmonary hypertension. Biomed Pharmacother. 2019; 109:1740–9. 10.1016/j.biopha.2018.11.058 30551428

[33] Shu R, Wong W, Ma QH, Yang ZZ, Zhu H, Liu FJ, Wang P, Ma J, Yan S, Polo JM, Bernard CC, Stanton LW, Dawe GS, Xiao ZC. APP intracellular domain acts as a transcriptional regulator of miR-663 suppressing neuronal differentiation. Cell Death Dis. 2015; 6:e1651. 10.1038/cddis.2015.10 25695604 PMC4669786

[34] Zhang Z, Ao P, Han H, Zhang Q, Chen Y, Han J, Huang Q, Huang H, Zhuo D. LncRNA PLAC2 upregulates miR-663 to downregulate TGF-β1 and suppress bladder cancer cell migration and invasion. BMC Urol. 2020; 20:94. 10.1186/s12894-020-00663-w 32650766 PMC7350696

[35] Li S, Lu X, Zheng D, Chen W, Li Y, Li F. Methyltransferase-like 3 facilitates lung cancer progression by accelerating m6A methylation-mediated primary miR-663 processing and impeding SOCS6 expression. J Cancer Res Clin Oncol. 2022; 148:3485–99. 10.1007/s00432-022-04128-5 35907010 PMC11800879

[36] Arora S, Dev K, Agarwal B, Das P, Syed MA. Macrophages: Their role, activation and polarization in pulmonary diseases. Immunobiology. 2018; 223:383–96. 10.1016/j.imbio.2017.11.001 29146235 PMC7114886

[37] Luo P, Qiu B. The role of immune cells in pulmonary hypertension: Focusing on macrophages. Hum Immunol. 2022; 83:153–63. 10.1016/j.humimm.2021.11.006 34844784

[38] Fan Y, Hao Y, Gao D, Li G, Zhang Z. Phenotype and function of macrophage polarization in monocrotaline-induced pulmonary arterial hypertension rat model. Physiol Res. 2021; 70:213–26. 10.33549/physiolres.934456 33676385 PMC8820576

[39] Wang Y, Zhao M, Liu S, Guo J, Lu Y, Cheng J, Liu J. Macrophage-derived extracellular vesicles: diverse mediators of pathology and therapeutics in multiple diseases. Cell Death Dis. 2020; 11:924. 10.1038/s41419-020-03127-z 33116121 PMC7595091

[40] Li C, Deng C, Zhou T, Hu J, Dai B, Yi F, Tian N, Jiang L, Dong X, Zhu Q, Zhang S, Cui H, Cao L, Shang Y. MicroRNA-370 carried by M2 macrophage-derived exosomes alleviates asthma progression through inhibiting the FGF1/MAPK/STAT1 axis. Int J Biol Sci. 2021; 17:1795–807. 10.7150/ijbs.59715 33994863 PMC8120458

[41] Guiot J, Cambier M, Boeckx A, Henket M, Nivelles O, Gester F, Louis E, Malaise M, Dequiedt F, Louis R, Struman I, Njock MS. Macrophage-derived exosomes attenuate fibrosis in airway epithelial cells through delivery of antifibrotic miR-142-3p. Thorax. 2020; 75:870–81. 10.1136/thoraxjnl-2019-214077 32759383 PMC7509395

[42] Yu F, Zhang X, Sun C, Xu W, Xia J. Downregulation of miRNA-663b protects against hypoxia-induced injury in cardiomyocytes by targeting BCL2L1. Exp Ther Med. 2020; 19:3581–8. 10.3892/etm.2020.8644 32346421 PMC7185160

[43] Huang X, Fan R, Lu Y, Yu C, Xu X, Zhang X, Liu P, Yan S, Chen C, Wang L. Regulatory effect of AMP-activated protein kinase on pulmonary hypertension induced by chronic hypoxia in rats: in vivo and in vitro studies. Mol Biol Rep. 2014; 41:4031–41. 10.1007/s11033-014-3272-9 24566685

[44] Dillard J, Meng X, Nelin L, Liu Y, Chen B. Nitric oxide activates AMPK by modulating PDE3A in human pulmonary artery smooth muscle cells. Physiol Rep. 2020; 8:e14559. 10.14814/phy2.14559 32914566 PMC7507575

[45] Sutendra G, Michelakis ED. The metabolic basis of pulmonary arterial hypertension. Cell Metab. 2014; 19:558–73. 10.1016/j.cmet.2014.01.004 24508506

[46] Klouda T, Yuan K. Inflammation in Pulmonary Arterial Hypertension. Adv Exp Med Biol. 2021; 1303:351–72. 10.1007/978-3-030-63046-1_19 33788202

[47] Dong H, Li X, Cai M, Zhang C, Mao W, Wang Y, Xu Q, Chen M, Wang L, Huang X. Integrated bioinformatic analysis reveals the underlying molecular mechanism of and potential drugs for pulmonary arterial hypertension. Aging (Albany NY). 2021; 13:14234–57. 10.18632/aging.203040 34016786 PMC8202883

[48] Moral-Sanz J, Lewis SA, MacMillan S, Meloni M, McClafferty H, Viollet B, Foretz M, Del-Pozo J, Mark Evans A. AMPK deficiency in smooth muscles causes persistent pulmonary hypertension of the new-born and premature death. Nat Commun. 2022; 13:5034. 10.1038/s41467-022-32568-7 36028487 PMC9418192

[49] Zhang Q, Li W, Zhu Y, Wang Q, Zhai C, Shi W, Feng W, Wang J, Yan X, Chai L, Chen Y, Li C, Liu P, Li M. Activation of AMPK inhibits Galectin-3-induced pulmonary artery smooth muscle cells proliferation by upregulating hippo signaling effector YAP. Mol Cell Biochem. 2021; 476:3037–49. 10.1007/s11010-021-04131-3 33797701

[50] Chen J, Zhang M, Liu Y, Zhao S, Wang Y, Wang M, Niu W, Jin F, Li Z. Histone lactylation driven by mROS-mediated glycolytic shift promotes hypoxic pulmonary hypertension. J Mol Cell Biol. 2022; mjac073. [Epub ahead of print]. 10.1093/jmcb/mjac073 36564027 PMC10175659

[51] Dai J, Zhou Q, Chen J, Rexius-Hall ML, Rehman J, Zhou G. Alpha-enolase regulates the malignant phenotype of pulmonary artery smooth muscle cells via the AMPK-Akt pathway. Nat Commun. 2018; 9:3850. 10.1038/s41467-018-06376-x 30242159 PMC6155017

[52] Achanta LB, Thomas DS, Housley GD, Rae CD. AMP-activated protein kinase activators have compound and concentration-specific effects on brain metabolism. J Neurochem. 2023. [Epub ahead of print]. 10.1111/jnc.15815 36977628

[53] Zhou J, Yan S, Guo X, Gao Y, Chen S, Li X, Zhang Y, Wang Q, Zheng T, Chen L. Salidroside protects pancreatic β-cells against pyroptosis by regulating the NLRP3/GSDMD pathway in diabetic conditions. Int Immunopharmacol. 2023; 114:109543. 10.1016/j.intimp.2022.109543 36508922

[54] Brittain EL, Niswender K, Agrawal V, Chen X, Fan R, Pugh ME, Rice TW, Robbins IM, Song H, Thompson C, Ye F, Yu C, Zhu H, et al. Mechanistic Phase II Clinical Trial of Metformin in Pulmonary Arterial Hypertension. J Am Heart Assoc. 2020; 9:e018349. 10.1161/JAHA.120.018349 33167773 PMC7763730

[55] Gui D, Cui Z, Zhang L, Yu C, Yao D, Xu M, Chen M, Wu P, Li G, Wang L, Huang X. Salidroside attenuates hypoxia-induced pulmonary arterial smooth muscle cell proliferation and apoptosis resistance by upregulating autophagy through the AMPK-mTOR-ULK1 pathway. BMC Pulm Med. 2017; 17:191. 10.1186/s12890-017-0477-4 29233105 PMC5726034

[56] Yang D, Sun C, Zhang J, Lin S, Zhao L, Wang L, Lin R, Lv J, Xin S. Proliferation of vascular smooth muscle cells under inflammation is regulated by NF-κB p65/microRNA-17/RB pathway activation. Int J Mol Med. 2018; 41:43–50. 10.3892/ijmm.2017.3212 29115381 PMC5746293

[57] Xiao Q, Zhang S, Yang C, Du R, Zhao J, Li J, Xu Y, Qin Y, Gao Y, Huang W. Ginsenoside Rg1 Ameliorates Palmitic Acid-Induced Hepatic Steatosis and Inflammation in HepG2 Cells via the AMPK/NF-κB Pathway. Int J Endocrinol. 2019; 2019:7514802. 10.1155/2019/7514802 31467529 PMC6699274

[58] Sung JY, Kim SG, Kim JR, Choi HC. Prednisolone suppresses adriamycin-induced vascular smooth muscle cell senescence and inflammatory response via the SIRT1-AMPK signaling pathway. PLoS One. 2020; 15:e0239976. 10.1371/journal.pone.0239976 32997729 PMC7526920

[59] Zhai C, Shi W, Feng W, Zhu Y, Wang J, Li S, Yan X, Wang Q, Zhang Q, Chai L, Li C, Liu P, Li M. Activation of AMPK prevents monocrotaline-induced pulmonary arterial hypertension by suppression of NF-κB-mediated autophagy activation. Life Sci. 2018; 208:87–95. 10.1016/j.lfs.2018.07.018 30009823

[60] Zurlo G, Piquereau J, Moulin M, Pires Da Silva J, Gressette M, Ranchoux B, Garnier A, Ventura-Clapier R, Fadel E, Humbert M, Lemaire C, Perros F, Veksler V. Sirtuin 1 regulates pulmonary artery smooth muscle cell proliferation: role in pulmonary arterial hypertension. J Hypertens. 2018; 36:1164–77. 10.1097/HJH.0000000000001676 29369849

[61] Xi L, Ruan L, Yao X, Zhang D, Yuan H, Li Q, Yan C. SIRT1 promotes pulmonary artery endothelial cell proliferation by targeting the Akt signaling pathway. Exp Ther Med. 2020; 20:179. 10.3892/etm.2020.9309 33101469 PMC7579766

[62] Chen HZ, Wang F, Gao P, Pei JF, Liu Y, Xu TT, Tang X, Fu WY, Lu J, Yan YF, Wang XM, Han L, Zhang ZQ, et al. Age-Associated Sirtuin 1 Reduction in Vascular Smooth Muscle Links Vascular Senescence and Inflammation to Abdominal Aortic Aneurysm. Circ Res. 2016; 119:1076–88. 10.1161/CIRCRESAHA.116.308895 27650558 PMC6546422

[63] Han L, Zhang Y, Zhang M, Guo L, Wang J, Zeng F, Xu D, Yin Z, Xu Y, Wang D, Zhou H. Interleukin-1β-Induced Senescence Promotes Osteoblastic Transition of Vascular Smooth Muscle Cells. Kidney Blood Press Res. 2020; 45:314–30. 10.1159/000504298 32126555

[64] Ruderman NB, Xu XJ, Nelson L, Cacicedo JM, Saha AK, Lan F, Ido Y. AMPK and SIRT1: a long-standing partnership? Am J Physiol Endocrinol Metab. 2010; 298:E751–60. 10.1152/ajpendo.00745.2009 20103737 PMC2853213

[65] Xu C, Song Y, Wang Z, Jiang J, Piao Y, Li L, Jin S, Li L, Zhu L, Yan G. Pterostilbene suppresses oxidative stress and allergic airway inflammation through AMPK/Sirt1 and Nrf2/HO-1 pathways. Immun Inflamm Dis. 2021; 9:1406–17. 10.1002/iid3.490 34342160 PMC8589405

[66] Zhang CY, Hu XC, Zhang GZ, Liu MQ, Chen HW, Kang XW. Role of Nrf2 and HO-1 in intervertebral disc degeneration. Connect Tissue Res. 2022; 63:559–76. 10.1080/03008207.2022.2089565 35736364

[67] Mansouri A, Reiner Ž, Ruscica M, Tedeschi-Reiner E, Radbakhsh S, Bagheri Ekta M, Sahebkar A. Antioxidant Effects of Statins by Modulating Nrf2 and Nrf2/HO-1 Signaling in Different Diseases. J Clin Med. 2022; 11:1313. 10.3390/jcm11051313 35268403 PMC8911353

[68] Lv H, Zhu C, Wei W, Lv X, Yu Q, Deng X, Ci X. Enhanced Keap1-Nrf2/Trx-1 axis by daphnetin protects against oxidative stress-driven hepatotoxicity via inhibiting ASK1/JNK and Txnip/NLRP3 inflammasome activation. Phytomedicine. 2020; 71:153241. 10.1016/j.phymed.2020.153241 32454347

[69] He Y, Zhong JH, Wei XD, Huang CY, Peng PL, Yao J, Song XS, Fan WL, Li GC. Pachymic Acid Ameliorates Pulmonary Hypertension by Regulating Nrf2-Keap1-ARE Pathway. Curr Med Sci. 2022; 42:56–67. 10.1007/s11596-021-2414-2 34881424

[70] Sevilla-Montero J, Munar-Rubert O, Pino-Fadón J, Aguilar-Latorre C, Villegas-Esguevillas M, Climent B, Agrò M, Choya-Foces C, Martínez-Ruiz A, Balsa E, Muñoz-Calleja C, Gómez-Punter RM, Vázquez-Espinosa E, et al. Cigarette smoke induces pulmonary arterial dysfunction through an imbalance in the redox status of the soluble guanylyl cyclase. Free Radic Biol Med. 2022; 193:9–22. 10.1016/j.freeradbiomed.2022.09.026 36174878

[71] Pan J, Wang R, Pei Y, Wang D, Wu N, Ji Y, Tang Q, Liu L, Cheng K, Liu Q, Sun J, Gong M, Zheng X, et al. Sulforaphane alleviated vascular remodeling in hypoxic pulmonary hypertension via inhibiting inflammation and oxidative stress. J Nutr Biochem. 2023; 111:109182. 10.1016/j.jnutbio.2022.109182 36220525

