# Supplementary material for: Exosomal miR-663b from “M1” macrophages promotes pulmonary artery vascular smooth muscle cell dysfunction through inhibiting the AMPK/Sirt1 axis
Source: Aging (Albany NY). 2023 May 4;15(9):3549–71. doi: 10.18632/aging.204690 (PMC10449306; doi:10.18632/aging.204690)
Supplement: Supplementary Figures [file aging-15-204690-s001.pdf]

SUPPLEMENTARY FIGURES

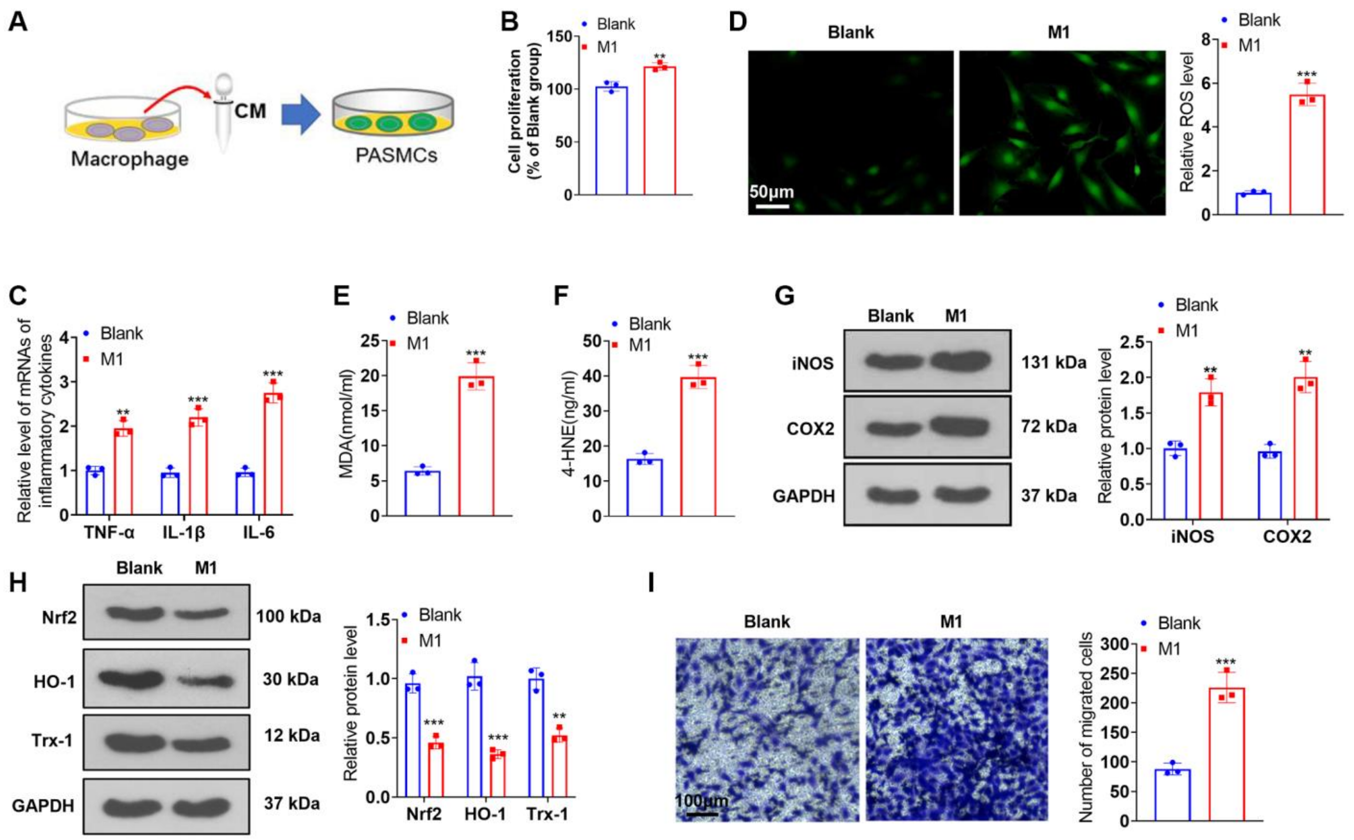

Supplementary Figure 1. M1 macrophages induced PASMC dysfunction. (**A**) A co-culture model of M1 macrophages and PASMCs was built. (**B**) CCK8 assay was used for evaluating PASMC proliferation. (**C**) RT-PCR was done for ascertaining the levels of inflammatory cytokines in PASMCs. (**D**–**F**) Cell immunofluorescence and colorimetry determined the levels of ROS, MDA, and 4-HNE in PASMCs. (**G**, **H**) Western blot measured iNOS, COX2, Nrf2, HO-1 and Trx-1 levels in PASMCs. (**I**) Transwell assay monitored PASMC migration. *N* = 3. $^{**}P < 0.01$, $^{***}P < 0.001$ (vs. Blank).

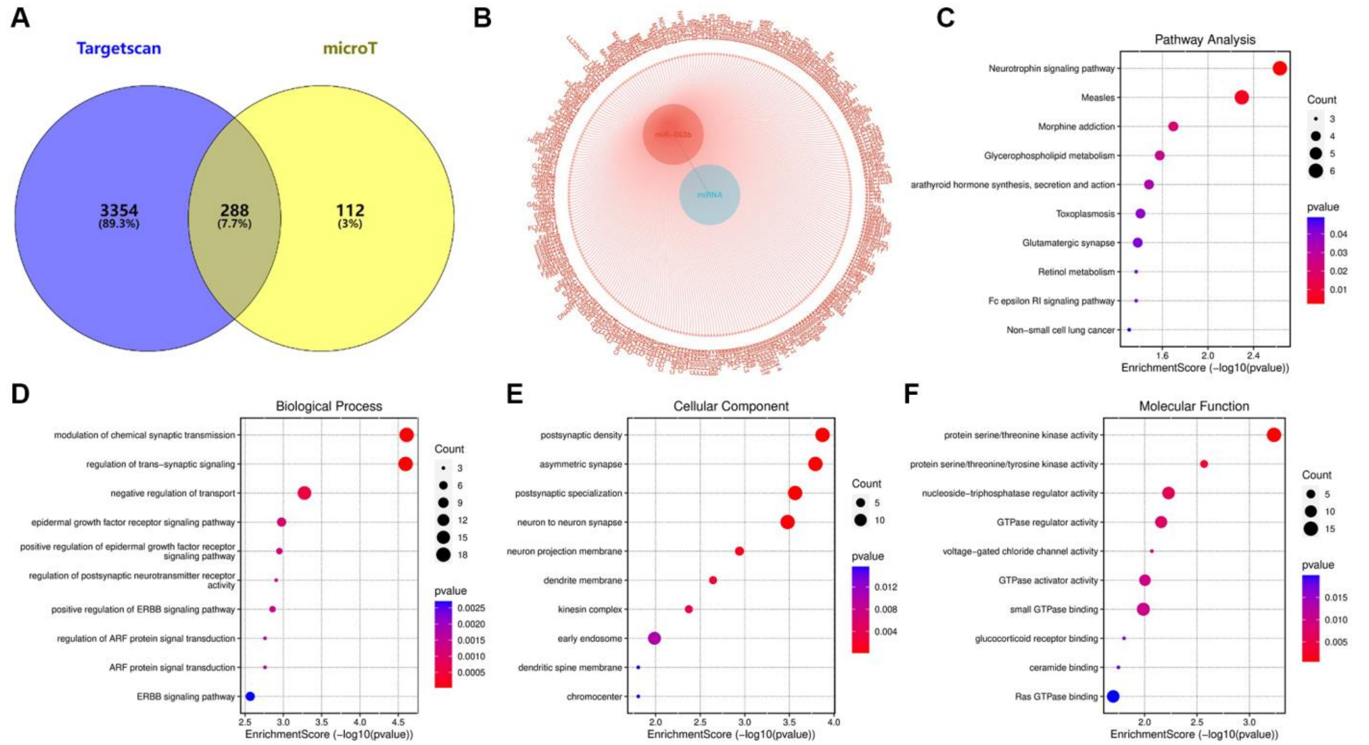

Supplementary Figure 2. The bioinformatic analysis of the target of miR-663b. (**A**) The targets of miR-663b were analyzed through Targetscan database (https://www.targetscan.org/vert_72/) and microT databse (https://dianalab.e-ce.uth.gr/html/dianauniverse/index.php?r=microT_CDS). (**B**) The miRNA-gene target network was shown. (**C**–**F**) Gene enrichment analysis was performed through the online database DAVID (https://david.ncifcrf.gov/home.jsp). The enriched KEGG pathways and biological themes, particularly GO terms (including biological process (BP), cellular component (CC), and molecular function (MF)) were shown.
